# microRNAs’ differential regulations mediate the progress of Human Papillomavirus (HPV)-induced Cervical Intraepithelial Neoplasia (CIN)

**DOI:** 10.1186/s12918-015-0145-3

**Published:** 2015-02-07

**Authors:** Wenjuan Mo, Chao Tong, Yan Zhang, Hong Lu

**Affiliations:** State Key Laboratory of Genetic Engineering, School of Life Science, and Institute of Biomedical Sciences, Fudan University, 200433 Shanghai, China; Department of Gynaecology and Obstetrics in Changhai Hospital, 200433 Shanghai, China; Shanghai Engineering Research Center of Industrial Microorganisms, 200433 Shanghai, China; Collaborative Innovation Center of Cancer Medicine, 200433 Shanghai, China

**Keywords:** microRNA’s differential regulation, Regulatory network construction, Cervical intraepithelial neoplasia

## Abstract

**Background:**

microRNA (miRNA)’s direct regulation on target mRNA is affected by complex factors beyond miRNA. Therefore, at different stages during the course of carcinogenesis, miRNA may regulate different targets, which we termed ‘miRNA’s differential regulation’. HPV-induced cervical intraepithelial neoplasia (CIN) is an important pre-cancerous course ahead of cervical cancer formation. Currently, the molecular mechanisms of CIN progress remain poorly understood, and it is interesting to unravel this from the perspective of miRNA differential regulation.

**Results:**

In this study, we performed transcriptome analysis of miRNAs and mRNAs for the totally 24 cervical samples in three stages (normal, CIN I, and CIN III) along CIN progress, and proposed the SIG++ algorithm to detect the miRNA — mRNA pairs with significant regulation change, and further proposed the definitions of Efficient Pair, Efficient Target, and Related Effector Biological Process, as the elemental steps to construct miRNA differential regulatory network. Finally, for the course of disease progressing from normal stage to CIN I stage, and for the course of disease progressing from CIN I stage to CIN III stage, miRNA differential regulatory networks were constructed, respectively, based on two distinct strategies: one is founded on the knowledge of human GO biological processes to detect Efficient Targets and Related Effector Biological Processes, the other is solely founded on literature review to detect the targets closely related to cervical carcinogenesis and instructive in revealing mechanisms that promote CIN development.

**Conclusions:**

This study provided the conception of miRNA’s differential regulation, the algorithm for how to identify them during disease development, and the strategy for how to construct miRNA differential regulatory network with instructive biological meanings. The finally constructed networks provide clues for understanding CIN progress.

**Electronic supplementary material:**

The online version of this article (doi:10.1186/s12918-015-0145-3) contains supplementary material, which is available to authorized users.

## Background

In animal cells, microRNA (miRNA) usually targets mRNA’s 3′ untranslated region (3′ UTR) through its 5′ seed (2 nt ~ 7 nt) pairing, resulting in mRNA destabilization or translational repression. Based on sequence prediction, a miRNA may have binding sites on hundreds of mRNAs, and an mRNA’s 3′UTR may also be bound by tens of miRNAs (miRanda [1]; miRDB [2]; etc.). Besides, miRNA’s direct binding on target mRNA may be affected by other complex factors [3], thus during carcinogenesis progress, a miRNA may regulate different target mRNAs in different stages. For instance, along prostate cancer development, we previously identified that miR-133b represses the cell cycle control proteins RB1CC1 and PTPRK at the early androgen-dependent stage [4], thus essentially promotes cell proliferation; while another group identified miR-133b functions as tumor repressor by targeting epidermal growth factor receptor (EGFR) at the late androgen-independent stage [5]. Likewise, during breast cancer development, miR-224 inhibits cell invasion at the early non-invasive stage by targeting chemokine receptor CXCR4 and cell division cycle protein CDC42 [6]; while at the late invasive stage, miR-224 promotes tumor cell’s bone metastasis by targeting phosphatidylethanolamine binding protein RKIP [7]. Therefore, the phenomenon of miRNA regulating different targets at different stages may be universal, and we term it ‘microRNA’s differential regulation’. Systematically studying this aspect during tumor formation, i.e. to find miRNA’s specific targets at each stage, is critical for integrally understanding carcinogenesis mechanisms.

A miRNA’s regulation on an mRNA can be mainly represented by their negatively correlated co-expression, since miRNAs usually cause target mRNA degradation via exonucleases or P-body [8]. Currently, there are several studies analyzing the significant change of mRNA — mRNA co-expression during cancer progress [9,10], which partially reflects the change of protein — protein interaction. To our knowledge, to date there is no report for systematically studying the significant change of miRNA — mRNA co-expression along disease development, as well as how to precisely determine the cellular processes influenced by miRNA’s differential regulation.

Cervical cancer is one of the most lethal gynecologic malignancy, and is prevalently caused by human papillomavirus (HPV) infection. HPV intrudes into cervix through tiny wound and infects the basal cells in epithelium. In most cases the infection is cleared by the immune system, and in some cases the infection persists and leads the mild cervical lesions to progress through cervical intraepithelial neoplasia (CIN). During CIN progress, CIN I, CIN II, and CIN III are the early, middle, and late stages, respectively, referring to the lesions happened in the lower third of, two-thirds of, and more than two-thirds of epithelium, respectively. After CIN III stage, malignant cells invade into the pelvic cavity, thus cervical cancer forms. In the field of genome-wide research for cervical carcinogenesis [11-14], current works mainly focus on cervical cancer, and analyzing the role of certain miRNA with significant expression change between tumor and normal tissue. Since CIN is the important pre-cancerous course with many uncertainties surrounding its natural history, it is necessary to perform genome-wide miRNA and mRNA expression analysis along CIN stages, and reveal the potential mechanisms from the new perspective of miRNA differential regulation.

In this study, we proposed SIG++ algorithm as well as subsequent analysis strategy, to systematically construct miRNA differential regulation mediated networks along CIN progress. 7 normal cervical samples (HPV-), 9 CIN I samples (HPV+), and 8 CIN III samples (HPV+) were conducted with miRNA and mRNA expression microarray analysis, respectively. The finally constructed networks provide mechanism clues for gradual cellular deterioration and HPV ultimate integration during CIN progress.

## Methods

**Figure 1:**
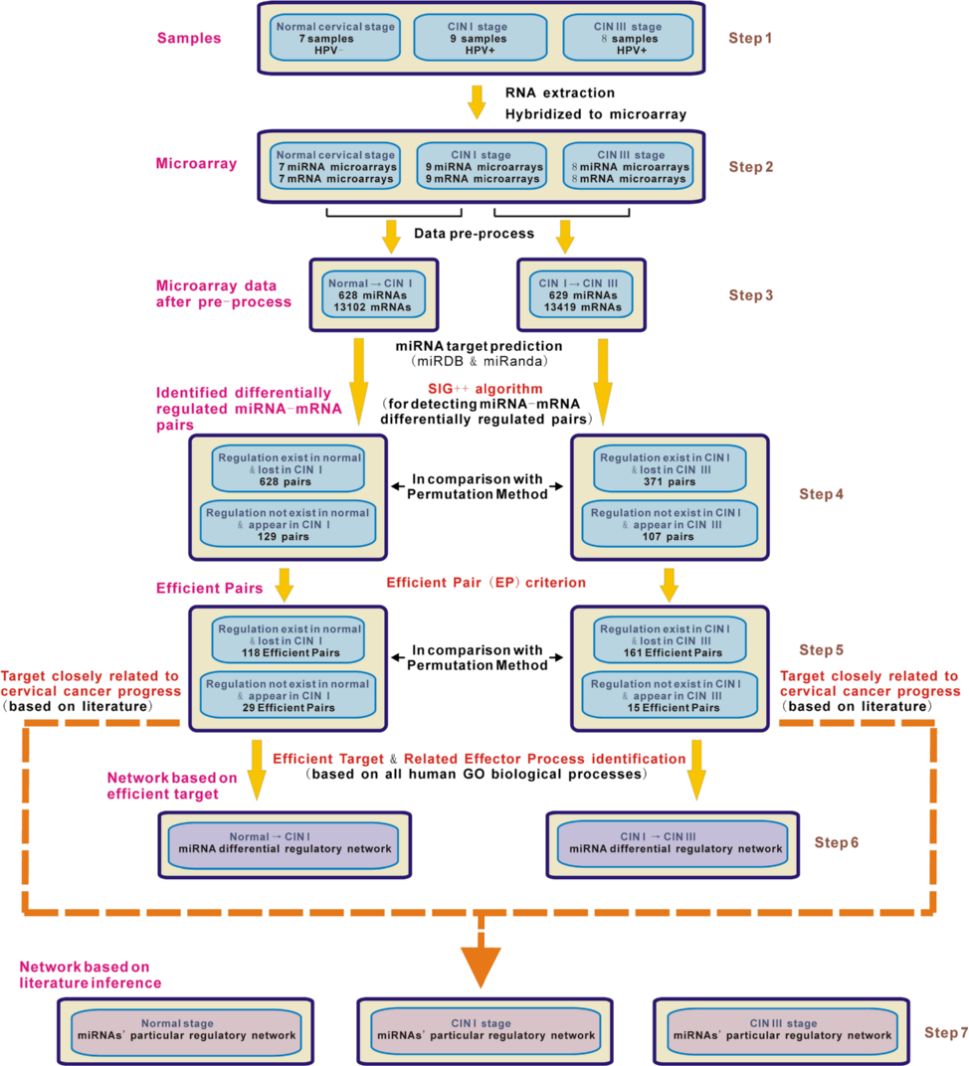
**Flowchart of strategy.** This is the outline for analysing microarray data to construct miRNAs’ differential regulation network in this study. Detailed steps are provided in the method and result sections.

The flowchart of strategy for constructing miRNA differential regulation mediated networks in this study is presented in Figure 1. The involved materials and methods are described stepwise in the following.

### Ethics statement

At the first diagnosis, patients provided written consent that their cervical tissue samples could be used for investigational purposes. Institutional approval from local research ethical committee was obtained for the conduct of the study (Department of Gynaecology and Obstetrics in Changhai Hospital, Shanghai). Samples were analyzed anonymously.

### Clinical samples

This is Step 1 in the flowchart (Figure 1). Each cervical sample was obtained from a different patient in the Gynaecology and Obstetrics Department of Changhai Hospital, and was partitioned into two sections instantly: one put into liquid nitrogen for research use, the other subsequently analyzed with histopathological CIN grading and HPV testing. Normal cervical sample refers to the tissue adjacent to early lesions and with no HPV detection. Then for each sample, its total RNA was extracted and purified using the RNeasy Mini Kit (Qiagen, Inc., Valencia, California). Only when its RNA quality met the criteria for microarray analysis, the sample was selected for research. Thus after careful specimen collection, 7 normal cervical samples (HPV-), 9 CIN I samples (HPV+), and 8 CIN III samples (HPV+) were used for microarray analysis.

### Genome-wide expression profile by Illumina BeadArray

This is Step 2 in the flowchart (Figure 1). Total RNAs in each sample were hybridized to Illumina HumanHT-12 V4.0 expression beadchip (for mRNA) and Illumina Human v2 MicroRNA expression beadchip (for miRNA) separately. The raw data were uploaded to the Gene Expression Omnibus public repository (http://www.ncbi.nlm.nih.gov/geo/; Gene Expression Omnibus series no. GSE51993).

### Data pre-processing

This is Step 3 in the flowchart (Figure 1). For microarray data of miRNA and mRNA expressions, we used cubic spline method for normalization, respectively. Then we used previous method to amend the unauthentic data [4]. Next, we performed gene filtering, based on the abundance of unauthentic data. For a gene, we set the number of its unauthentic data in normal, CIN I, and CIN III samples as *n*_N_, *n*_I_, and *n*_III_, respectively. The microarray data were partitioned into two parts: normal vs. CIN I, and CIN I vs. CIN III. For the data of normal vs. CIN I, a gene was excluded if *n*_N_ ≥ 3 or *n*_I_ ≥ 4; for the data of CIN I vs. CIN III, a gene was excluded if *n*_I_ ≥ 4 or *n*_III_ ≥ 4. Afterwards, the whole data were regarded as credible.

### Prediction of miRNA’s target mRNA based on sequence

Before identifying the miRNA — mRNA pairs with significant regulation change, we first obtain the information of miRNAs’ potential target mRNAs based on in silico sequence prediction. The miRDB database [2] and miRanda database [1] were used for miRNA target prediction in this study. The human miRNA targets were downloaded from miRDB version 4.0 database, in which all targets were predicted by a tool MirTarget2, which was developed with an SVM learning machine. Also, the targets of conserved human miRNAs with good mirSVR score were downloaded from miRanda August 2010 release. Then for a miRNA, the targets both predicted by the databases of miRDB and miRanda, were used for analyzing differential regulation in this study.

### SIG++ algorithm for identifying miRNA — mRNA pair with significant regulation change during CIN progress

This is Step 4 in the flowchart (Figure 1). miRNA’s regulation on target mRNA can be mainly represented by the Pearson correlation coefficient *r* [8]. If *r* < 0, it is regarded that miRNA has regulation on mRNA. During disease progress from the earlier stage (e.g. normal stage) to the later stage (e.g. CIN I stage), for a miRNA — mRNA pair, if miRNA’s regulation on mRNA exists in normal but does not exist in CIN I, or does not exist in normal but exists in CIN I, it is termed ‘regulation change’ or ‘differential regulation’.

In order to find the predicted miRNA — mRNA pairs with significant regulation change, based on the SIG (Stochastic process model for Identifying differentially co-expressed Gene pair) algorithm [10], we present the SIG++ algorithm in this study as follows. For a miRNA, we calculate the Pearson correlation coefficient *r* of this miRNA with its predicted target mRNA at one stage. Then transform *r* into a metric *z* using Fisher’s *r*-to-*z* transformation as *z* = ln[(1 + *r*)/(1 - *r*)]/2. The *z*-transformed correlation coefficient is labeled as *z*-CC, which is normally distributed. For a miRNA, let *X* and *Y* denote the cohort of *z*-CCs for all predicted miRNA — mRNA pairs at two stages, respectively, with both *X* and *Y* normally distributed:

$$ \begin{array}{c}\hfill {f}_X(x)=\frac{1}{\sqrt{2\uppi}{\upsigma}_{\mathrm{X}}} \exp \left[-\frac{{\left(\mathrm{x}-{\upmu}_{\mathrm{X}}\right)}^2}{2{\upsigma}_{\mathrm{X}}^2}\right]\hfill \\ {}\hfill {f}_Y(y)=\frac{1}{\sqrt{2\uppi}{\upsigma}_{\mathrm{Y}}} \exp \left[-\frac{{\left(\mathrm{y}-{\upmu}_{\mathrm{Y}}\right)}^2}{2{\upsigma}_{\mathrm{Y}}^2}\right]\hfill \end{array}, $$

where *x* and *y* are the values of *X* and *Y*, while *μ*_*X*_, *μ*_*Y*_, *σ*_*X*_, *σ*_*Y*_ are the expectations and standard deviations, calculated according to clinical data.

Let *T* = *X*/*Y,* describing the co-expression change of a miRNA — mRNA pair. Its analytical distribution is:

$$ {f}_T(t)={\displaystyle {\int}_{-\infty}^{\infty }{f}_{XY}\left(x,y\right)\left|y\right| dy=}{\displaystyle {\int}_0^{\infty }{f}_{XY}\left(\mathrm{t}\mathrm{y},y\right)ydy-}{\displaystyle {\int}_{-\infty}^0{f}_{XY}\left(ty,y\right)ydy}, $$

where *fXY(x,y)* is the joint probability density of *X* and *Y*. z-CC started from the initial value at earlier stage and transformed to the finial value at later stage as disease progressed, indicating a causal association between initial and final values. Therefore, *fXY(x,y)* should be calculated by the conditional probability, which measures the probability for a variable developing to a certain final value when its initial value is given. There are two cases in the derivation of *f*_*T*_*(t)* based on the arrangements of *X* and *Y*.

1. *X* is the *z*-CC at later stage, and *Y* is the *z*-CC at earlier stage,
  $$ {f}_{XY}\left(x,y\right)=\mathrm{G}\left(\mathrm{x}\Big|\mathrm{y}\right){f}_Y(y), $$
  where G(x│y) is the conditional probability density for *z*-CC transforming from *y* into *x*. G(x│y)can be approximated using the solution of random walk model below.
  A random walk is a mathematical formalization of a path that consists of a succession of random steps [15]. Academically speaking, ‘random walk’ usually refers to the simple symmetric random walk on a locally finite lattice, with the same probabilities of the location jumping to each one of its immediate neighbours. In this study, the one-dimensional simple symmetric random walk model is used, and referred as ‘random walk model’ for simplicity. The random walk model can be illustrated by an elementary example for a particle: starts at 0 and at each step (the time interval between two steps is ∆τ) it moves ∆x or –∆x with equal probability, the probability of its being at position x after time τ is modeled as follows:
  $$ \overline{\upomega}\left(\mathrm{x},\uptau \right)=\frac{1}{2}\overline{\upomega}\left(\mathrm{x}-\Delta \mathrm{x},\uptau -\Delta \uptau \right)+\frac{1}{2}\overline{\upomega}\left(\mathrm{x}+\Delta \mathrm{x},\uptau -\Delta \uptau \right), $$
  where $\overline{\upomega}\left(\mathrm{x},\uptau \right)$ is the probability of the particle at *x* when the time point is τ. The solution of the above equation is approximated as
  $$ \mathrm{u}\approx {\mathrm{u}}_0\left(\mathrm{x},\uptau \right)\equiv {\left(4\uppi \mathrm{D}\uptau \right)}^{-\frac{1}{2}} \exp \left(-\frac{{\mathrm{x}}^2}{4\mathrm{D}\uptau}\right), $$
  where *u* is the probability density as $\mathrm{u}=\overline{\upomega}/2\varDelta \mathrm{x}$, and *D* is a constant as D = lim_*Δ*x → 0,*Δ*τ → 0_ (*Δ*x)^2^/2*Δ*x. That solution represents the probability of a variable under cumulative effect of many random events over the time interval τ to reach a final value when its initial value has been known. Based on this central idea, the random walk model is reminiscent of the conditional probability, since the cellular status during tumor formation can be regarded as varying randomly at each step along disease progress. Thus
  $$ \mathrm{G}\left(\mathrm{x}\Big|\mathrm{y}\right)\approx {\left(4\uppi \mathrm{D}\mathrm{d}\right)}^{-\frac{1}{2}} \exp \left[-\frac{{\left(\mathrm{x}-\mathrm{y}\right)}^2}{4\mathrm{D}\mathrm{d}}\right]. $$
  Finally, the analytical distribution of *T* is
  1 $$ {\mathrm{f}}_{\mathrm{T}}\left(\mathrm{t}\right)=\mathrm{K}\frac{1}{2\uppi \sqrt{2\mathrm{D}\mathrm{d}}{\upsigma}_{\mathrm{Y}}\mathrm{a}}{\mathrm{e}}^{-\mathrm{c}}\left[1-\mathrm{bAexp}\left(\frac{{\mathrm{b}}^2}{4\mathrm{a}}\right)\right], $$
  where
  $$ \mathrm{a}=\frac{{\left(\mathrm{t}-1\right)}^2}{4\mathrm{D}\mathrm{d}}+\frac{1}{2{\upsigma}_{\mathrm{Y}}^2},\cdot \cdot \mathrm{b}=-\frac{\upmu_{\mathrm{Y}}}{\upsigma_{\mathrm{Y}}^2},\cdot \mathrm{c}=\frac{\upmu_{\mathrm{Y}}^2}{2{\upsigma}_{\mathrm{Y}}^2}, $$
  and the integration $\mathrm{A}={\displaystyle {\int}_{\mathrm{b}/2\mathrm{a}}^0 \exp \left(-{\mathrm{ay}}^2\right)\mathrm{dy}}$ can be calculated by numerical integration. *K* is a normalization constant. *d* and *D* are constants and in general let *d* = *D* = 1.
2. *X* is the *z*-CC at earlier stage, and *Y* is the *z*-CC at later stage,
  $$ {f}_{XY}\left(x,y\right)=\mathrm{G}\left(\mathrm{y}\Big|\mathrm{x}\right){f}_X(x). $$
  Similarly, the derived analytical distribution of *T* is
  2 $$ {\mathrm{f}}_{\mathrm{T}}\left(\mathrm{t}\right)=\mathrm{K}\hbox{'}\frac{1}{2\uppi \sqrt{2\mathrm{D}\mathrm{d}}{\upsigma}_{\mathrm{X}}{\mathrm{a}}^{\hbox{'}}}{\mathrm{e}}^{-{\mathrm{c}}^{\hbox{'}}}\left[1-\mathrm{b}\hbox{'}\mathrm{A}\hbox{'} \exp \left(\frac{{\mathrm{b}\hbox{'}}^2}{4\mathrm{a}\hbox{'}}\right)\right], $$
  where
  $$ \mathrm{a}\hbox{'}=\frac{{\left(\mathrm{t}-1\right)}^2}{4\mathrm{D}\mathrm{d}}+\frac{1}{2{\upsigma}_{\mathrm{X}}^2},\cdot \cdot \mathrm{b}\hbox{'}=-\frac{{\mathrm{t}\upmu}_{\mathrm{X}}}{\upsigma_{\mathrm{X}}^2},\cdot {c}^{\prime }=\frac{\upmu_{\mathrm{x}}^2}{2{\upsigma}_{\mathrm{x}}^2}, $$
  and the integration $\mathrm{A}`={\displaystyle {\int}_{\mathrm{b}/2\mathrm{a}}^0 \exp \left(-\mathrm{a}'{\mathrm{y}}^2\right)\mathrm{dy}}$. *K*’ is a normalization constant, and *d* = *D* = 1.
  For a miRNA, based on its analytical distribution *f*_*T*_*(t)* (see Additional file 1, the diagrammatic sketch), we can detect which target mRNAs have significant regulation change with it during disease progress. Set the threshold value α for significance as 0.05. For a miRNA — mRNA pair, if its clinical ratio value t^*^ lies in the shadow region (see Additional file 1), i.e. *p* < 0.05, this pair is regarded with significant regulation change.
  The procedure of SIG++ algorithm is summarized in the following. For a miRNA, we first identify the miRNA — mRNA pairs with regulation nonexistent in earlier stage (stage 1) and existent in later stage (stage 2):
  - According to Equation (1), numerically calculate the theoretical co-expression change distribution *f*_*T*_*(t)* for this miRNA based on it all predicted target mRNAs.
  - Calculate the Pearson coefficients *r*_1_ and r_2_ of this miRNA with a target mRNA at stage 1 and stage 2, respectively. Note that noise is treated here: if −0.2 < *r* < 0, it accounts for little correlation, the sign is caused by noise, thus let *r* = |*r*|. Then perform *z*-transformation, and calculate the ratio t^*^ = *z*_2_/*z*_1_. Finally based on *f*_*T*_*(t)*, calculate the *p* value. If *p* < 0.05, this pair is recognized with little regulation at stage 1, and strong regulation at stage 2.
    To obtain the miRNA — mRNA pairs with regulation existent at stage 1, and nonexistent at stage 2, we repeat the above steps a ~ b. Note that now in step a, *f*_*T*_*(t)* is calculated based on Equation (2), and in step b, the ratio t^*^ = *z*_1_/*z*_2_.
    For the normal, CIN I, and CIN III stages, we repeat the above procedure, then obtain the whole repertoire of miRNA — mRNA pairs with significant regulation change between adjacent stages.

### Permutation method for identifying miRNA — mRNA pair with significant regulation change during CIN progress

For comparing the results obtained by the SIG++ algorithm, we also used the permutation method, which is the traditional statistic method to assess the significance *p* value, to identify miRNA — mRNA pairs with significant regulation change between two stages. The procedure is carried out as follows. In each permutation, randomly pick up a miRNA and an mRNA (regardless of sequence prediction), mix their expression data in two stages, and then randomly distribute the mixed data into two stages for miRNA and mRNA, respectively. After that, calculate the co-expression ratio value *t* for this pair. Perform such permutation 10^6^ times to obtain the background distribution for measuring the significance of clinical miRNA — mRNA regulation change.

The *p* value for a clinically calculated value t^*^ is estimated in the following. Sort the 10^6^ permutated *t* values in ascending order. If t^*^ < 0, then

$$ \mathrm{p}=\frac{\mathrm{n}\left\{\mathrm{t}<{\mathrm{t}}^{*}\right\}}{10^6}; $$

if t^*^ > 0, then

$$ \mathrm{p}=\frac{\mathrm{n}\left\{\mathrm{t}>{\mathrm{t}}^{*}\right\}}{10^6}, $$

where *n*{*t* < t^*^} and *n*{*t* > t^*^} denote the number of *t* values less than t^*^and larger than t^*^, respectively.

If *p* < 0.05, then the corresponding miRNA — mRNA pair is considered with significant regulation change.

### Criterion of efficient pair

This is Step 5 in the flowchart (Figure 1). Among the predicted miRNA — mRNA pairs with significant regulation change identified either by the SIG++ algorithm or by the Permutation method, we further proposed the criterion of ‘Efficient miRNA — mRNA differential regulation Pair’, or simply termed ‘Efficient Pair’, to select the pairs with moderate reliability for subsequent analysis. The definition of ‘Efficient Pair’ is based on the accordance between pair’s regulation change and expression change, described as follows. In this study, for a miRNA or an mRNA, the obvious expression change (upregulated or downregulated) between earlier stage and later stage is defined as:

$$ \frac{\left|{\overline{a}}_L-{\overline{a}}_E\right|}{{\overline{a}}_E}>30\%, $$

where *ā*_*E*_ and *ā*_*L*_ are the average expression value of this gene in earlier and later stages, respectively. As listed in Table 1, for a miRNA — mRNA pair, we defined the agreement or disagreement between its regulation change and expression change, and correspondingly defined ‘Agreement Pair’ (AP) and ‘Typical Pair’ (TP). AP and TP are collectively termed ‘Efficient Pair’ (EP). For a pair detected as EP, it is regarded that its regulation change and expression change are in accordance, thus this pair has moderate reliability prepared for the subsequent network construction.

**Table 1:** **Definition of the accordance between regulation change and expression change for a miRNA — mRNA pair**

**Regulation in stage 1**	**Regulation in stage 2**	**miRNA change**	**mRNA change**	**Relationship definition**	**Pair definition**
Exist	Not exist	↘	↗	Agreement	Agreement Pair (AP)
Exist	Not exist	↗	↘	Disagreement	—
Exist	Not exist	-	↗	—	Typical Pair (TP)
Not exist	Exist	↗	↘	Agreement	Agreement Pair (AP)
Not exist	Exist	↘	↗	Disagreement	—
Not exist	Exist	-	↘	—	Typical Pair (TP)

In this table, for a miRNA — mRNA pair, its regulation change, miRNA expression change and mRNA expression change between stage 1 (earlier stage) and stage 2 (later stage) are listed, thereupon the accordance between regulation change and expression change is defined. ‘miRNA change’ or ‘mRNA change’: the expression change of miRNA or mRNA; ‘↗’ or ‘↘’ : the expression was upregulated or downregulated in stage 2 compared with stage 1; ‘-’: the expression has no obvious change between two stages. ’Agreement’ or ‘Disagreement’: for a miRNA — mRNA pair, its regulation change and expression change is in agreement or in disagreement. The ‘Agreement Pair’ (AP) and ‘Typical Pair’ (TP) are collectively termed ‘Efficient Pair’ (EP).

### Pathway enrichment of efficient pairs

To analyze the pathway enrichment of efficient pairs, or more precisely speaking, to analyze the pathway enrichment of targets involved in the efficient miRNA — mRNA pairs, we used the MAS system provided by CapitalBio company, which is a freely available website (Molecule Annotation System, http://bioinfo.capitalbio.com/mas3/), and the output results are considerably reliable since it covers all the knowledge of Gene Ontology (GO), KEGG, GenMapp, and Biocarta during calculation.

### Identification of efficient target & related effector biological process

For the above detected efficient pairs, we further attempt to identify the ‘Efficient Target’ whose contribution to a certain GO biological process is altered according to miRNAs’ differential regulation, thus mediating miRNA’s differential regulation to the effector process. In this study, for an mRNA gene i involved in a GO biological process k, its contribution to this GO process is measured by the Efficient Score (e_ik_):

$$ {\mathrm{e}}_{\mathrm{ik}}=\frac{{\displaystyle {\sum}_{\mathrm{j}=1}^{{\mathrm{N}}_{\mathrm{k}}}}{\mathrm{r}}_{\mathrm{ij}}}{{\mathrm{N}}_{\mathrm{k}} - 1},\cdot {\mathrm{N}}_{\mathrm{k}}\ge 2,\cdot \mathrm{i}\ne \mathrm{j}, $$

where N_k_ is the total number of genes in process k, r_ij_ is the Pearson correlation coefficient for the expressions of gene i and gene j. e_ik_, the average of correlation coefficients of gene i with other genes in process k, can concisely manifest gene i’s involvement in this process. The larger the e_ik_, the stronger contribution of gene i on GO process k.

To estimate the significance *p* value of an Efficient Score, we performed 10^5^ permutations. In each permutation, mix the samples in normal, CIN I and CIN III stages, then randomly select 10 samples, as well as randomly select 10 genes which are assumed to form a fictitious GO process, and calculate the Efficient Score value e of an arbitrary gene in the fictitious process. After that, obtain 10^5^ permutated Efficient Scores, which work as the background distribution to assess the p value of an Efficient Score e^*^ calculated by clinical data. The significance p value is estimated as

$$ \mathrm{p}=\frac{\mathrm{n}\left\{\mathrm{e}>{\mathrm{e}}^{*}\right\}}{10^5}, $$

where *n*{e > e^*^} denotes the number of permutated Efficient Scores larger than e^*^. For an e_ik_, if p < 0.05, then gene i is regarded as having significant contribution to the GO process k; else, no contribution to the process.

Combining the knowledge of efficient pair and efficient score, we select the Efficient Target as well as the Related Effector Biological Process, which together present the significant downstream effect on cellular process that was mainly caused by miRNA’s differential regulation. The definition of Efficient Target and Related Effector Process is depicted in Table 2, based on the accordance of miRNA regulation change and mRNA contribution change. In other words, for an Efficient Pair, if the target has contribution to a biological process when it is not regulated by the miRNA, meanwhile the target has no contribution to the process when it is repressed by the miRNA, this target gene is termed ‘Efficient Target’, and the corresponding GO process is termed ‘Related Effector Biological Process’. Since the Efficient Target mediates miRNA’s differential regulation to the Related Effector Process, the miRNA differential regulatory network can be finally constructed, manifested by the streams of ‘miRNA → mRNA → cellular biological process’.

**Table 2:** **Definition of efficient target and related effector process**

**miRNA’s regulation in stage 1**	**miRNA’s regulation in stage 2**	**Target’s contribution to a process in stage 1**	**Target’s contribution to a process in stage 2**	**Target definition**	**GO process definition**
Exist	Not exist	Not exist	Exist	Efficient target	Related effector process
Not exist	Exist	Exist	Not exist	Efficient target	Related effector process

In this table, for an already detected Efficient Pair, we further analyze the accordance of miRNA regulation and target mRNA contribution to a process. As mentioned in context, the target’s contribution to a biological process is measured by the Efficient Score, if the Efficient Score is significant (p < 0.05), the mRNA’s contribution to the process is regarded as ‘exist’; elsewise, as ‘not exist’. For an Efficient Pair, if in one stage, miRNA’s repression on mRNA exists while mRNA’s contribution to the process does not exist; and in the other stage, miRNA’s repression on mRNA does not exist while mRNA’s contribution to the process exists, this target mRNA is defined as ‘Efficient Target’, and the corresponding process is defined as ‘Related Effector Process’. In summary, the Related Effector Process is significantly influenced by miRNA’s differential regulation, through the Efficient Target mediation.

In this study, all the Homo sapiens GO biological processes with involved genes were downloaded from Amigo2 website (http://amigo.geneontology.org/amigo). There are totally 9,710 Human GO processes. Note that the GO biological process containing at least 2 pre-processed microarray genes and at least one gene involved in the Efficient Pair, is used for Efficient Score calculation.

### Construction of miRNA differential regulatory network

Founded on the detected Efficient Pairs in Step 5 (Figure 1), we used two distinct strategies to construct miRNA differential regulatory networks. One (Step 6) is based on known GO biological processes, through identifying Efficient Target & Related Effector Process; the other (Step 7) is solely based on literature review, including the latest reports closely related to cervical carcinogenesis, to detect the target genes with potential key roles in promoting CIN progress.

## Results and discussion

### SIG++ algorithm identifies miRNA — mRNA pairs with differential regulation during CIN progress

**Figure 2:**
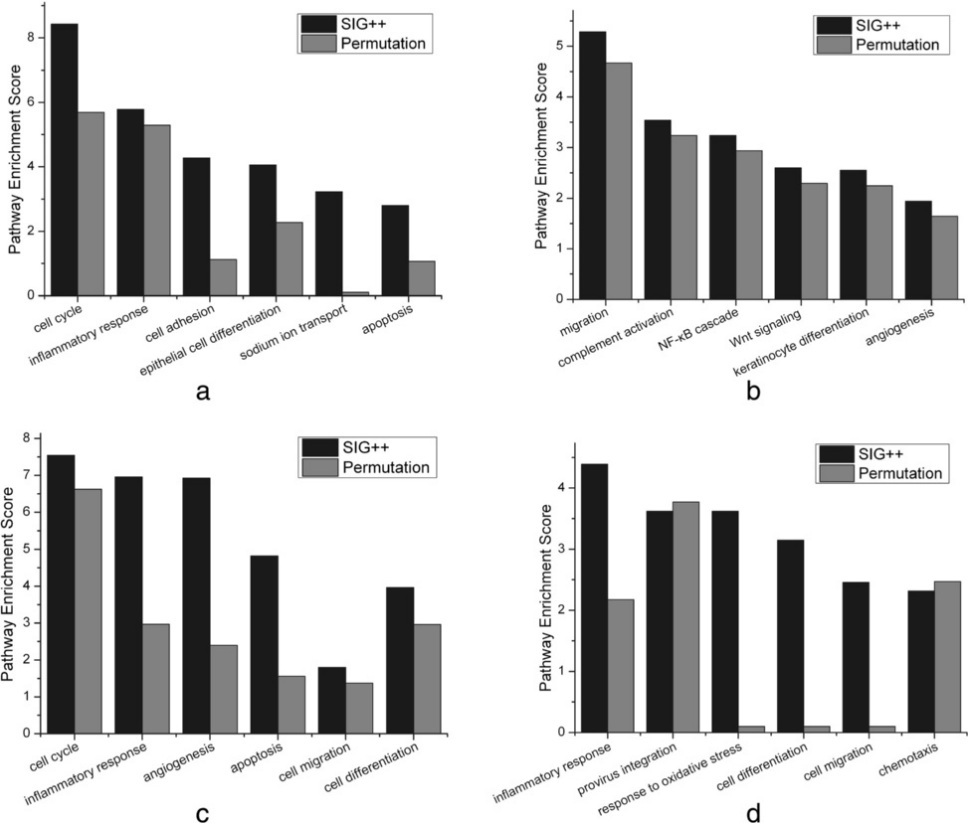
**Comparison of pathway enrichment for Efficient Pairs detected by the two methods.** The Efficient Pairs (EPs) were identified by the SIG++ method and by the Permutation method, respectively, and the pathway enrichment analysis for the involved targets are illustrated in the sub-figures (a ~ d). **(a)** EPs with regulation existent in normal and nonexistent in CIN I; **(b)** EPs with regulation nonexistent in normal and existent in CIN I; **(c)** EPs with regulation existent in CIN I and nonexistent in CIN III; **(d)** EPs with regulation nonexistent in CIN I and existent in CIN III. In each sub-figure, Pathway Enrichment Score = −lg*P*, where *P* refers to the enrichment significance of a biological process.

We performed miRNA and mRNA expression microarray analysis for 7 normal cervical samples (HPV-), 9 CIN I samples (HPV+), and 8 CIN III samples (HPV+), respectively. After data pre-process, 628 miRNAs and 13102 mRNAs remained for the progress from normal to CIN I, 629 miRNAs and 13419 mRNAs remained for the progress from CIN I to CIN III. We obtained all predicted miRNA — mRNA pairs by combining miRDB and miRanda databases. Next, we applied the SIG++ method and permutation method to the microarray data, respectively, and detected the miRNA — mRNA pairs with significant regulation change. We compared the two methods’ results from two aspects: one is the number of identified pairs, including the Efficient Pair, as listed in Table 3; the other is the pathway enrichment for Efficient Pairs, as illustrated in Figure 2.

**Table 3:** **Comparison of SIG++ method and permutation method from the number of identified miRNA — mRNA pairs**

**Data**	**Cases of differential regulation**	**Number of miRNA—** **mRNA Pairs**	**Methods**	
			**SIG++**	**Permutation**
**Normal vs. CIN I**	**Regulations exist in normal & not exist in CIN I**	*N*_total_	401	181
		*N*_AP_	37	12
		*N*_TP_	81	39
		*N*_EP_ = *N*_AP_ + *N*_TP_	118(Set 1)	51
	**Regulations not exist in normal & exist in CIN I**	*N*_total_	129	266
		*N*_AP_	20	38
		*N*_TP_	9	15
		*N*_EP_ = *N*_AP_ + *N*_TP_	29 (Set 2)	53
**CIN I vs. CIN III**	**Regulations exist in CIN I & not exist in CIN III**	*N*_total_	371	93
		*N*_AP_	109	37
		*N*_TP_	52	10
		*N*_EP_ = *N*_AP_ + *N*_TP_	161 (Set 3)	47
	**Regulations not exist in CIN I & exist in CIN III**	N_total_	107	65
		N_AP_	6	3
		N_TP_	9	6
		N_EP_ = N_AP_ + N_TP_	15 (Set 4)	9

This table lists the number of miRNA — mRNA pairs with significant regulation change detected by the SIG++ method and by the Permutation method, respectively. N_total_: the number of detected differentially regulated miRNA — mRNA pairs whose targets have obvious expression change; N_AP_: the number of Agreement Pair (AP); N_TP_: the number of Typical Pair (TP); N_EP_: the number of Efficient Pair (EP). For the Efficient Pairs detected by the SIG++ algorithm, ‘Set 1’ refers to the set of EP whose regulation exist in normal stage and not exist in CIN I stage, ‘Set 2’ refers to the set of EP with regulation nonexistent in normal and existent in CIN I, ‘Set 3’ refers to the set of EP with regulation existent in CIN I and nonexistent in CIN III, and ‘Set 4’ refers to the set of EP with regulation nonexistent in CIN I and existent in CIN III.

As shown in Table 3, in comparison with the results identified by the Permutation method, for the progress from normal stage to CIN I stage, the SIG++ method identified larger number of efficient miRNA — mRNA pairs with regulation existent in normal and nonexistent in CIN I, while identified less number of efficient pairs with regulation nonexistent in normal and existent in CIN I; for the progress from CIN I stage to CIN III stage, the SIG++ method identified both larger number of the efficient pairs, either with regulation existent in CIN I and nonexistent in CIN III, or with regulation nonexistent in CIN I and existent in CIN III. On the whole, the SIG++ method can detect larger amounts of Efficient Pairs and therefore has the potential to reveal the molecular mechanisms of CIN progress more comprehensively.

As illustrated in Figure 2, for both the two methods, their identified Efficient Pairs are enriched in the pathways highly related to CIN progress, such as cell cycle, apoptosis, migration, angiogenesis, and inflammation, etc. Furthermore, in comparison with the Permutation method, the Efficient Pairs detected by the SIG++ method are much higher-enriched in the key pathways (Figure 2). The above comparison suggest that the SIG++ algorithm may be more competent in inferring the essential pathways significant influenced by miRNAs’ differential regulation.

### Pathway enrichment analysis of miRNA regulation existence along CIN progress generalize the characteristics for each stage

**Figure 3:**
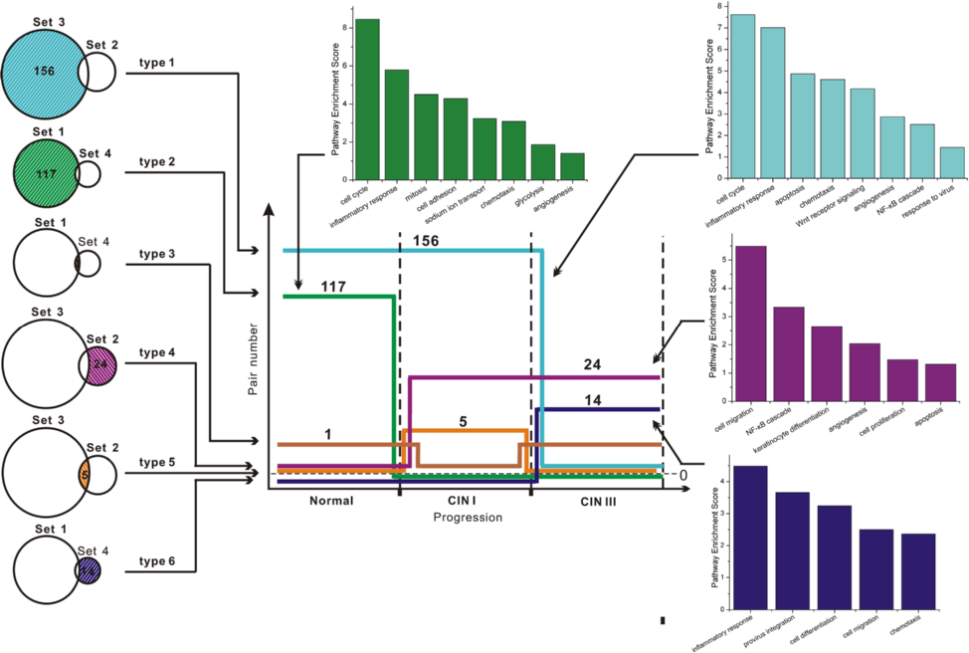
**Pathway enrichment of specially existent miRNA — mRNA regulation for each stage along CIN progress.** For the Efficient Pairs (EPs) detected by the SIG++ method, the central part in this figure displays the particular existence of miRNA — mRNA regulations along the stages. Each color has its own meaning exhibited visually in the figure and is correspondingly mentioned in the context, such as ‘Green’ referring to the Efficient Pairs with miRNA regulation only existent in normal stage. The left part (i.e. Venn diagram) in this figure shows how to partition the Efficient Pairs into 6 types. The meanings of Set 1 ~ Set 4 are described in Table 3, and their shadow sections in Venn diagram refer to the EPs with regulation particularly existent in certain stage(s), indicated in corresponding colors. Finally, the pathway enrichment of EPs with particularly existent regulation in certain stage(s) are also illustrated in corresponding colors.

Based on the Efficient Pairs detected by the SIG++ algorithm, we are further curious to know the characteristic pathways involved in miRNA regulation at each stage along CIN progress. To achieve this, we analyzed the pathway enrichment of the Efficient Pairs whose regulation particularly exist in the interested stage. For the sets of Efficient Pairs (Table 3, Set 1 ~ Set 4), there are 6 types of intersection (Figure 3, shown by Venn diagram), which represent the miRNA — mRNA pairs with regulation existent only in normal stage (Figure 3, type 2), or CIN I stage (type 5), or CIN III stage (type 6), respectively, and also represent the miRNA — mRNA pairs with regulation existent both in normal and CIN I stages (type 1), or both in CIN I and CIN III stages (type 4), or both in normal and CIN III stages (type 3), respectively. The pathway enrichment analysis of EPs in each intersection type illustrate that 117 pairs only have regulation in normal stage (Figure 3, indicated in green), with targets enriched in the processes of inflammation, cell adhesion, glycolysis, angiogenesis, etc.; 5 pairs only have regulation in CIN I stage (Figure 3, orange); 14 pairs only have regulation in CIN III stage (Figure 3, navy blue), with targets enriched in provirus integration, cell differentiation, cell migration, etc.; 156 pairs have regulation both in normal and CIN I stages (Figure 3, light blue), with targets enriched in inflammation, apoptosis, Wnt signaling, angiogenesis, etc.; 1 pair has regulation both in normal and CIN III stages (Figure 3, brown); 24 pairs have regulation both in CIN I and CIN III stages (Figure 3, purple), with targets enriched in keratinocyte differentiation, cell proliferation, cell migration, NF-κB signaling, etc. To sum up, for each stage, the specifically existent miRNA — mRNA regulations are enriched in inflammation; for normal stage, the specific miRNA regulations are particularly enriched in sodium ion transport and glycolysis; since CIN I stage, miRNA regulations have been highly enriched in cell migration and differentiation; finally for CIN III stage, the specific miRNA regulations are particularly enriched in virus integration.

### Construction of miRNA differential regulatory network based on efficient targets

Founded on the 323 Efficient Pairs identified by the SIG++ algorithm (Table 3, Set 1 ~ Set 4), we further calculated the Efficient Scores for the targets involved, and thereupon identified the Efficient Targets as well as the Related Effector Processes (defined in the method section), to finally construct miRNAs’ differential regulatory networks for the progress from normal stage to CIN I stage, and for the progress from CIN I stage to CIN III stage, respectively. The detailed results in this strategy of network construction are presented stepwise in the following.

**Figure 4:**
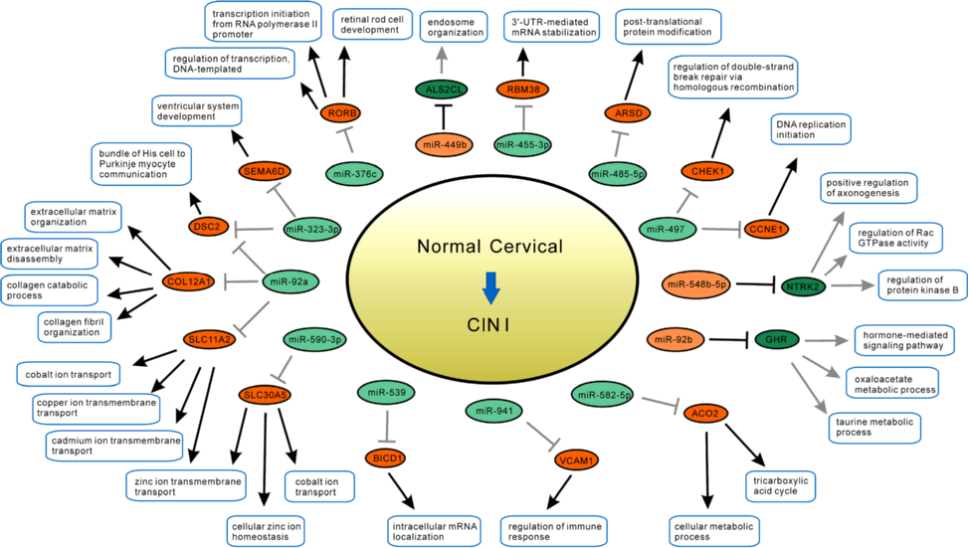
**miRNA differential regulatory network for disease progressing from normal stage to CIN I stage.** This network was constructed based on Efficient Target and Related Effector Biological Process. Note that either for miRNA’s repression on target mRNA, or for mRNA’s contribution to GO biological process, the black line denotes the action exists in the later stage (CIN I stage) but not in the earlier stage (normal stage), while the grey line denotes the action exists in the earlier stage but not in the later stage. Both for miRNAs and mRNAs, the red color denotes the expression is upregulated, and the green color denotes the expression is downregulated.

**Figure 5:**
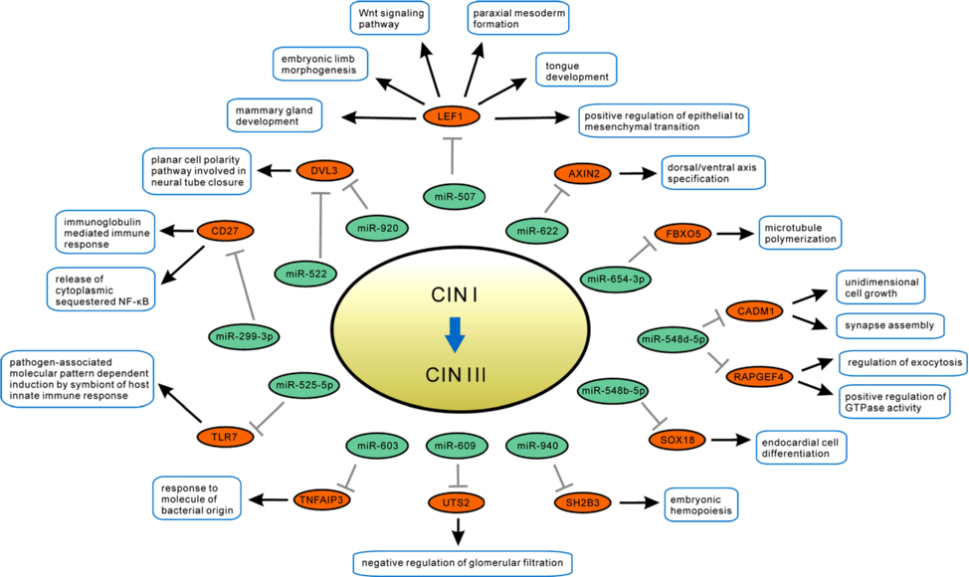
**miRNA differential regulatory network for disease progressing from CIN I stage to CIN III stage.** This network was also constructed based on Efficient Target and Related Effector Biological Process. The colors have the same meaning as in Figure 4. Note that here the earlier and later stages refer to the CIN I stage and CIN III stage, respectively.

There were totally 9710 Human GO biological processes provided in the Amigo2 website. For each of these processes, we downloaded the gene names contained, and then matched the microarray genes remained after data pre-process in this study with the downloaded gene names, thus obtained the information of GO biological processes containing the pre-processed microarray genes. After that, the GO process containing at least 2 pre-processed microarray genes and at least one gene involved in Efficient Pair, was used for Efficient Score calculation. For the progress from normal stage to CIN I stage, there were 993 GO processes remained for Efficient Score calculation, and thereafter 16 Efficient Targets and 28 Related Effector Processes were identified; for the progress from CIN I stage to CIN III stage, 1,016 GO processes remained for Efficient Score calculation, and 12 Efficient Targets and 20 Related Effector Processes were identified. Based on these results, miRNA differential regulatory networks systematically constituted by the streams of ‘miRNA → Efficient Target → Related Effector Process’ were constructed as illustrated in Figure 4 (for progress from normal to CIN I) and Figure 5 (for progress from CIN I to CIN III), respectively. The functional interpretation of these networks are provided below.

For disease progressing from normal stage to CIN I stage, there were totally 13 miRNAs, 16 Efficient Targets, and 28 Related Effector Processes involved in the network (Figure 4).

For the efficient target ACO2, miR-582-5p repressed it in normal stage but not in CIN I stage, thereby ACO2 having contribution to the GO processes of ‘tricarboxylic acid cycle’ and ‘cellular metabolic process’ in CIN I stage. In details, ACO2 encodes an enzyme that catalyzes the interconversion of citrate to isocitrate via cis-aconitate in the second step of the TCA cycle, which can promote tumor cell growth and proliferation [16]. This suggests that through miR-582-5p’s differential regulation on ACO2, the TCA cycle may be accelerated in CIN I stage, thus providing sufficient fuels for cell activity.

For the efficient target GHR, miR-92b repressed it only in CIN I stage, thus it had influence to the processes of ‘oxaloacetate metabolic process’, ‘taurine metabolic process’, and ‘hormone-mediated signaling pathway’ only in normal stage. In details, GHR encodes a member of the type I cytokine receptor family. It has been reported that taurine has repression effect on cervical cancer cell growth [17]. Therefore, we infer that through miR-92b’s differential regulation on GHR, taurine metabolic process may be enhanced in normal stage, thus arresting cell grow in normal stage; while in CIN I, the arrest on growth may be cancelled.

For the efficient target ALS2CL, miR-449b repressed it only in CIN I stage, and it had influence on the ‘endosome organization’ process only in normal stage. ALS2CL can dominantly suppress the endosome enlargement [18]. While endosome plays an important role during endocytosis, which is the main way of HPVs entering host cells [19]. Our finding suggests that through miR-449b’s differential regulation on ALS2CL, the enlargement of endosome was not constrained in CIN I stage, thus promoting HPV entrance into cells to form repeated infection.

For the efficient target ARSD, miR-485-5b repressed it only in normal stage, and it had contribution to the ‘post-translational protein modification’ process only in CIN I stage. ARSD encodes a member in the sulfatase family. ARSD and other sulfatase family enzymes are capable of hydrolyzing sulfonates produced by the cytosolic sulfotransferases (SULT) [20]. It is well known that dermatin sulfate and keratin sulfate are widely contained in the skin, and keratin sulfate is necessary for the keratocyte [21]. Our finding suggests that based on miR-485-5b’s differential regulation on ARSD, the degradation of keratin sulfate was enhanced in CIN I stage, thus promoting keratocyte pathological transformation.

For the efficient target BICD1, miR-539 repressed it only in normal stage, and it had contribution to the ‘intracellular mRNA localization’ process only in CIN I stage. BICD1 has been implicated in the transport from the Golgi apparatus to the endoplasmic reticulum. A study to investigate the link between vacuolar traffic and telomere length maintenance in Saccharomyces cerevisiae suggested that vacuolar genes probably regulate telomere length by effects on the telomerase and Ku pathways [22]. Based on this, a recent study also suggested shorter telomeres is associated with significantly lower BICD1 mRNA level in human leukocytes [23]. Our finding suggests that elicited by miR-539’s differential regulation on BICD1, the length of telomeres may be prolonged in CIN I stage, thus promoting cell immortalization transformation.

For the efficient target NTRK2, miR-548-5b repressed it only in CIN I stage, and it had contribution to the processes of ‘positive regulation of axonogenesis’, ‘regulation of Rac GTPase activity’, and ‘regulation of protein kinase B signaling’ only in normal stage. NTRK2 encodes a member of the neurotrophic tyrosine receptor kinase (NTRK) family. It has been reported that Am80 induces neuronal differentiation by increasing NTRK2 expression [24]. Our finding suggests that through miR-548-5b’s differential regulation on NTRK2, cell differentiation may be impaired in CIN I stage, thus leading cell to the poorly differentiated carcinogenesis transformation.

For the efficient target CCNE1, miR-497 repressed it only in normal stage, and it had contribution to the ‘DNA replication initiation’ process only in CIN I stage. CCNE1 encodes cyclin E1, which allows G1/S passage. Our finding suggests that based on miR-497’s differential regulation on CCNE1, the cell cycle G1/S transition may be promoted in CIN I stage.

For the efficient target CHEK1, miR-497 repressed it only in normal stage, and it had contribution to the ‘regulation of double-strand break repair via homologous recombination’ process only in CIN I stage. The protein encoded by CHEK1 belongs to the Ser/Thr protein kinase family, and is required for checkpoint-mediated cell cycle arrest in response to DNA damage, by integrating signals from ATM and ATR, to phosphorylate CDC25A protein, thereby delaying cell cycle in response to double-strand DNA breaks. On the other hand, it has been recently reported that HPV genomes associate with BRD4 to replicate at fragile sites in the host genome by utilizing host DNA damage responses, thus greatly increasing the chances of HPV integration [25]. Our finding suggests that based on miR-497’s differential regulation on CHEK1, the homologous recombination repair for DNA double-strand break was stimulated in CIN I stage, which may enhance the chance of HPV integration.

For the efficient target RBM38, miR-455-3p repressed it only in normal stage, and it had contribution to the ‘3′-UTR-mediated mRNA stabilization’ process only in CIN I stage. The protein encoded by RBM38 is a RNA-binding protein (RBP), and can interact with uridine-rich regions near miRNA target sequences to block miRNA’s binding on target, thus protecting the expression of genes transcribed by p53 regulation [26]. Our finding suggests that based on miR-455-3p’s differential regulation on RBM38, the response for DNA double-strand break may be promoted in CIN I stage by carrying out p53 downstream processes, thus may promote HPV integration.

For the efficient target COL12A1, miR-92a repressed it only in normal stage, and it had contribution to the processes of ‘extracellular matrix organization’, ‘extracellular matrix disassembly’, ‘collagen catabolic process’, and ‘collagen fibril organization’ only in CIN I stage. COL12A1 encodes the alpha chain of type XII collagen, which is a homotrimer found in association with type I collagen, an association that is thought to modify the interactions between collagen I fibrils and the surrounding matrix. Our finding suggests that based on miR-92a’s differential regulation on COL12A1, the interaction between cell and surrounding matrix may be enhanced in CIN I stage to promote cell invasion.

For the efficient target DSC2, miR-323-3p and miR-92a repressed it only in normal stage, and it had contribution to the process of ‘bundle of His cell to Purkinje myocyte communication’ only in CIN I stage. The protein encoded by DSC2 is a calcium-dependent glycoprotein as a member of the desmocollin family, whose members are found primarily in epithelial cells and are required for cell adhesion and desmosome formation [27]. Our finding suggests that based on miRNAs’ differential regulation on DSC2, the intercellular communication and cell adhesion may be enhanced in CIN I stage.

For the efficient target SEMA6D, miR-323-3p repressed it only in normal stage, and it had contribution to the ‘ventricular system development’ process only in CIN I stage. SEMA6D has been demonstrated as critically involved in cardiac morphogenesis [28]. In gastric carcinoma, SEMA6D may play an important role in tumor angiogenesis [29]. Our finding suggests that induced by miR-323-3p’s differential regulation on SEMA6D, the angiogenesis may be enhanced in CIN I stage.

For the efficient target VCAM1, miR-941 repressed it only in normal stage, thus it had contribution to the ‘regulation of immune response’ process only in CIN I stage. VCAM1 encodes vascular cell adhesion molecule, which mediates leukocyte-endothelial cell adhesion and signal transduction in order to enhance inflammation in cervical tissue [30]. Our finding suggests that based on miR-941’s differential regulation on VCAM1, the leukocyte-endothelial cell adhesion may be promoted in CIN I stage, thus accelerating inflammation in local tissue.

For the efficient target SLC11A2, miR-92a repressed it only in normal stage, thus it had contribution to the processes of ‘zinc ion transmembrane transport’, ‘cobalt ion transport’, ‘copper ion transmembrane transport’, and ‘cadmium ion transmembrane transport’ only in CIN I stage. SLC11A2 encodes a solute carrier protein and is involved in iron absorption, while iron is essential for host cell synthesis of virions [31]. Our finding suggests that through miR-92a’s differential regulation on SLC11A2, the ion transport in cells may be enhanced in CIN I stage, which may promote virion synthesis.

For the efficient target SLC30A5, miR-590-3p repressed it only in normal stage, thus it had contribution to the processes of ‘zinc ion transmembrane transport’, ‘cellular zinc ion homeostasis’, and ‘cobalt ion transport’ only in CIN I stage. Similar to SLC11A2, our finding suggests that through miR-590-3p’s differential regulation on SLC30A5, the ion transport in cells may be enhanced in CIN I stage to promote virion synthesis.

For disease progressing from CIN I stage to CIN III stage, there were totally 12 miRNAs, 12 Efficient Targets, and 20 Related Effector Processes involved in the miRNA differential regulatory network (Figure 5).

For the efficient target AXIN2, miR-622 repressed it in CIN I stage but not in CIN III stage, and AXIN2 having contribution to the ‘dorsal/ventral axis specification’ process only in CIN III stage. AXIN2 encodes the Axin-related protein, which plays an important role in the regulation of the stability of beta-catenin in the Wnt signaling, and can promote cell proliferation in ovarian cancer [32]. Our finding suggests that due to miR-622’s differential regulation on AXIN2, the Wnt signaling may be stabilized in CIN III stage.

For the efficient target DVL3, miR-920 repressed it only in CIN I stage, and it had contribution to the ‘planar cell polarity pathway involved in neural tube closure’ process only in CIN III stage. DVL3 encodes a cytoplasmic phosphoprotein which can activate the Wnt/β-catenin signaling in cervical cancer [33]. Our finding suggests that miR-920’s differential regulation on DVL3 may contribute to activating the Wnt signalling in CIN III stage.

For the efficient target LEF1, miR-507 repressed it only in CIN I stage, and it had contribution to the processes of ‘Wnt signaling pathway’, ‘embryonic limb morphogenesis’, ‘positive regulation of epithelial to mesenchymal transition’, ‘mammary gland development’, ‘tongue development’, and ‘paraxial mesoderm formation’ only in CIN III stage. Based on this, our finding suggests that miR-507’s differential regulation on LEF1, may lead to cell poor differentiation in CIN III stage.

For the efficient target FBXO5, miR-654-3p repressed it only in CIN I stage, and it had contribution to the ‘microtubule polymerization’ process only in CIN III stage. Note that ‘microtubule polymerization’ is closely related to cell cycle. The protein encoded by FBXO5 is a subunit of protein ligase complex, which has anti-apoptotic and proliferative abilities in breast carcinoma [34]. On the other hand, the high expression of FBXO5 can promote genomic instability [35]. Our finding suggests that due to miR-654-3p’s differential regulation on FBXO5, the cell proliferation as well as genomic instability may be enhanced in CIN III stage.

For the efficient target SH2B3, miR-940 repressed it only in CIN I stage, and it had contribution to the ‘embryonic hemopoiesis’ process only in CIN III stage. Our finding suggests that due to miR-940’s differential regulation on SH2B3, the angiogenesis process may be promoted in CIN III stage.

For the efficient target SOX18, miR-548b-5p repressed it only in CIN I stage, and it had contribution to the ‘embryonic hemopoiesis’ process only in CIN III stage. SOX18 has a role in cervical cancer by facilitating angiogenesis [36]. Our finding suggests that based on miR-548b-5p’s differential regulation on SOX18, the angiogenesis may be enhanced in CIN III stage.

For the efficient targets CD27, TLR7 and TNFAIP3, miR-299-3p, miR-525-5p and miR-603 repressed them only in CIN I stage, respectively, and these targets have contribution to the processes of ‘immunoglobulin mediated immune response’, ‘release of cytoplasmic sequestered NF-kappaB’, ‘pathogen - associated molecular pattern dependent induction by symbiont of host innate immune response’, and ‘response to molecule of bacterial origin’ only in CIN III stage. Our findings suggest that due to miRNAs’ differential regulation on CD27, TLR7 and TNFAIP3, the proinflammation processes may be greatly intensified in CIN III stage.

In summary, mainly based on observing the cervical samples in normal stage, CIN I stage, and CIN III stage in this study, the constructed miRNA differential regulatory networks for the course from normal stage to CIN I stage (Figure 4), and for the course from CIN I stage to CIN III stage (Figure 5), respectively, combinatively implicate that the common characteristic for CIN I and CIN III stages is inflammation and angiogenesis, the specific characteristic for CIN I stage is cell invasion and virion synthesis, and for CIN III stage is the poor differentiation. These derivation are majorly consistent with the characteristics previously deduced by pathway enrichment (illustrated in Figure 3).

### Construction of miRNA particular regulatory network at each stage solely based on literature review

**Figure 6:**
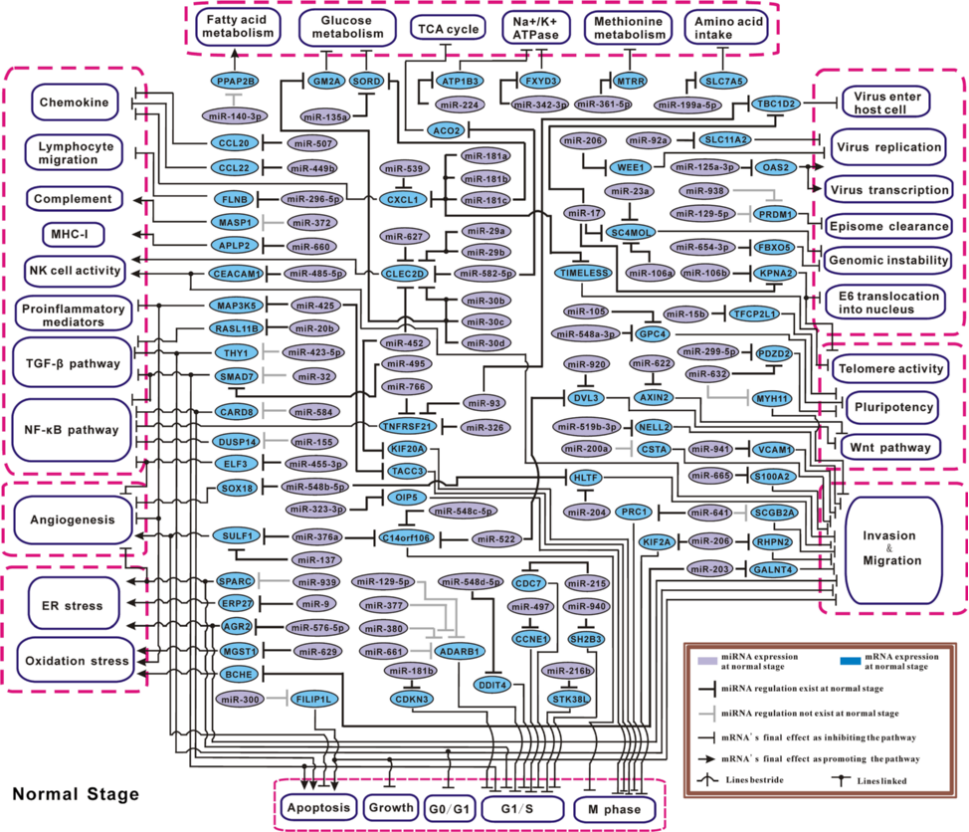
**miRNA particular regulatory network in normal stage.** This network and the following two networks were constructed based on literature inference. The symbol annotation is provided in the lower right corner in the figure. Note that, Purple/Blue: miRNA/mRNA expression at normal stage.

**Figure 7:**
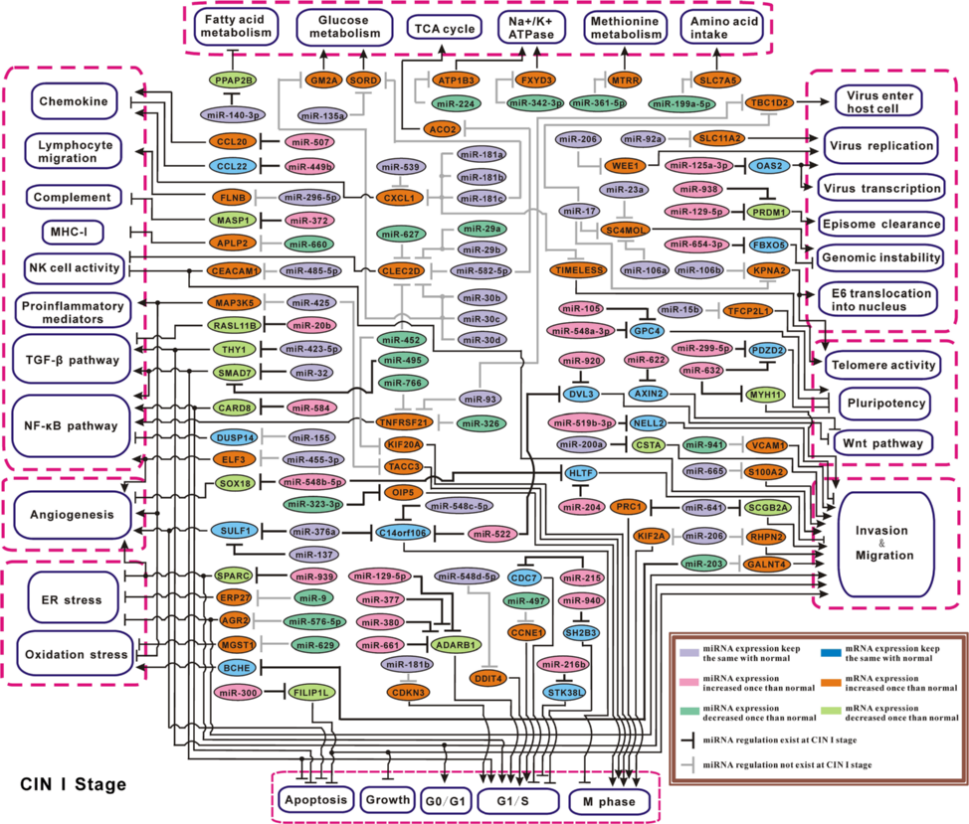
**miRNA particular regulatory network in CIN I stage.** Note that, Purple/Blue: miRNA/mRNA expression in CIN I stage has no obvious change compared with normal stage; Light pink/Orange: miRNA/mRNA expression in CIN I stage was upregulated than in normal stage, termed ‘increased once than normal’; Mint green/Grass green: miRNA/mRNA expression in CIN I stage was downregulated than in normal stage, termed ‘decreased once than normal’.

**Figure 8:**
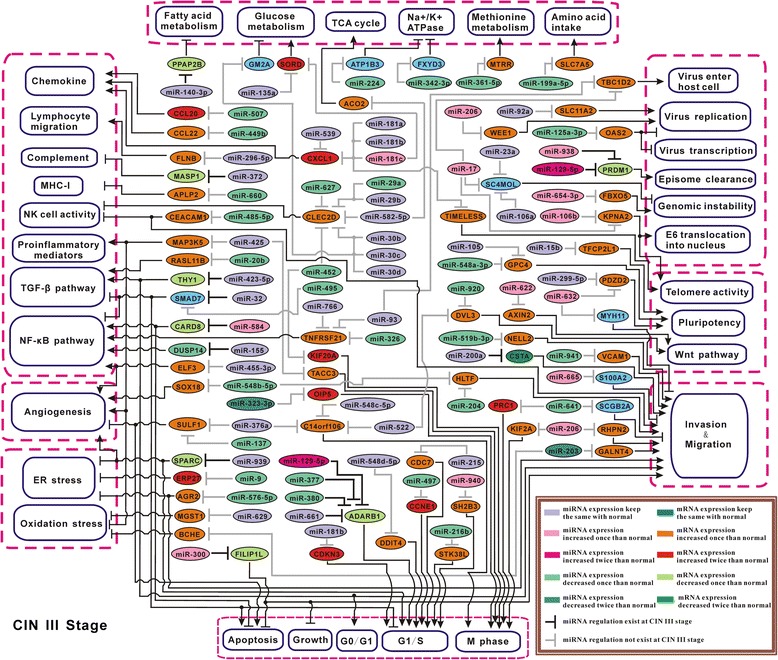
**miRNA particular regulatory network in CIN III stage.** Note that, Light pink/Orange: miRNA/mRNA expression in CIN III stage has no obvious change compared with CIN I stage when expression in CIN I stage was already increased, or miRNA/mRNA expression in CIN III stage was upregulated than CIN I stage when expression in CIN I stage had no obvious change compared with normal stage, termed ‘increased once than normal’; Magenta/Pure red: miRNA/mRNA expression in CIN III stage was upregulated than in CIN I stage when expression in CIN I stage was already increased, termed ‘increased twice than normal’. Likewise, the terms of ‘decreased once than normal’ and ‘decreased twice than normal’ have the analogic meanings.

On the other aspect, GO biological processes have been mainly summarized by manual review, which may have neglect on comprehensiveness and update, to overcome this, in this study we also used another way to construct miRNA differential regulatory network, by carrying out extensive literature review for the targets in the total 323 Efficient Pairs, to find genes closely related to cervical carcinogenesis. After that, we finally selected 99 Efficient Pairs, including 78 miRNAs and 69 target mRNAs. Based on those, we systematically constructed the miRNA particular regulatory networks for normal, CIN I, and CIN III stages, respectively (Figures 6, 7, and 8), displaying miRNAs’ regulation change and the possibly induced downstream effect from the eight aspects: inflammation, angiogenesis, cellular stress, apoptosis & proliferation, invasion & migration, virus infection, cell immortalization, and energy metabolism. See Additional file 2 for the detailed molecular elucidation for these networks (Figures 6, 7 and 8).

To generalize, during HPV-induced CIN progress, the malignant development in host cells has been promoted by multiple cooperative miRNA — mRNA regulation changes, as represented in the networks (Figures 4, 5, 6, 7 and 8), eventually leading to cervical cancer formation.

## Conclusions

In conclusion, we provided a genome-wide clinical data concerning miRNA and mRNA expressions for the three stages in HPV-induced CIN progress (normal, CIN I, and CIN III). By applying the proposed SIG++ algorithm, we obtained the miRNA — mRNA pairs with significant regulation change between two adjacent stages, and detected the Efficient Pairs among them, based on which we finally constructed the miRNA differential regulation networks for the CIN course, either from the perspective of Efficient Target and Related Effector Process, or from the perspective of sole literature review. To summarize, our work provides an effective strategy for studying miRNAs’ differential regulation during carcinogenesis development; also by analyzing the natural history of CIN, our work provides several molecular clues for therapeutic intervention of CIN regression.

### Availability of supporting data

The raw microarray data provided in this study are available in the Gene Expression Omnibus public repository (http://www.ncbi.nlm.nih.gov/geo/; Gene Expression Omnibus series no. GSE51993).

## Additional files

Additional file 1:
**Diagrammatic sketch of the analytical distribution function** ***fT(t).*** Description: *t* represents the co-expression change of a miRNA — mRNA pair between earlier stage and later stage. If the clinical value *t* ^*^ locates in the shadow region (*p* < 0.05), the corresponding pair is regarded as having significant regulation change between the two stages. Additional file 2:
**Molecular elucidation of miRNA particular regulatory networks constructed solely based on literature review.** This additional file provides detailed molecular elucidation for miRNA particular regulatory networks (Figures 6, 7, and 8) constituted by the 99 Efficient Pairs selected based on extensive literature review. Through our findings and inference, the potential molecular mechanisms for the course of CIN deterioration are provided from eight aspects: inflammation, angiogenesis, cellular stress, apoptosis & proliferation, invasion & migration, virus infection, cell immortalization, and energy metabolism.

## Abbreviations

CIN: Cervical intraepithelial neoplasia
SIG: Stochastic process model for identifying differentially co-expressed gene pair
AP: Agreement pair
TP: Typical pair
EP: Efficient pair
