# Supplementary figures and images for: microRNAs’ differential regulations mediate the progress of Human Papillomavirus (HPV)-induced Cervical Intraepithelial Neoplasia (CIN)
Source: BMC Syst Biol. 2015 Feb 7;9:4. doi: 10.1186/s12918-015-0145-3 (PMC4337110; doi:10.1186/s12918-015-0145-3)

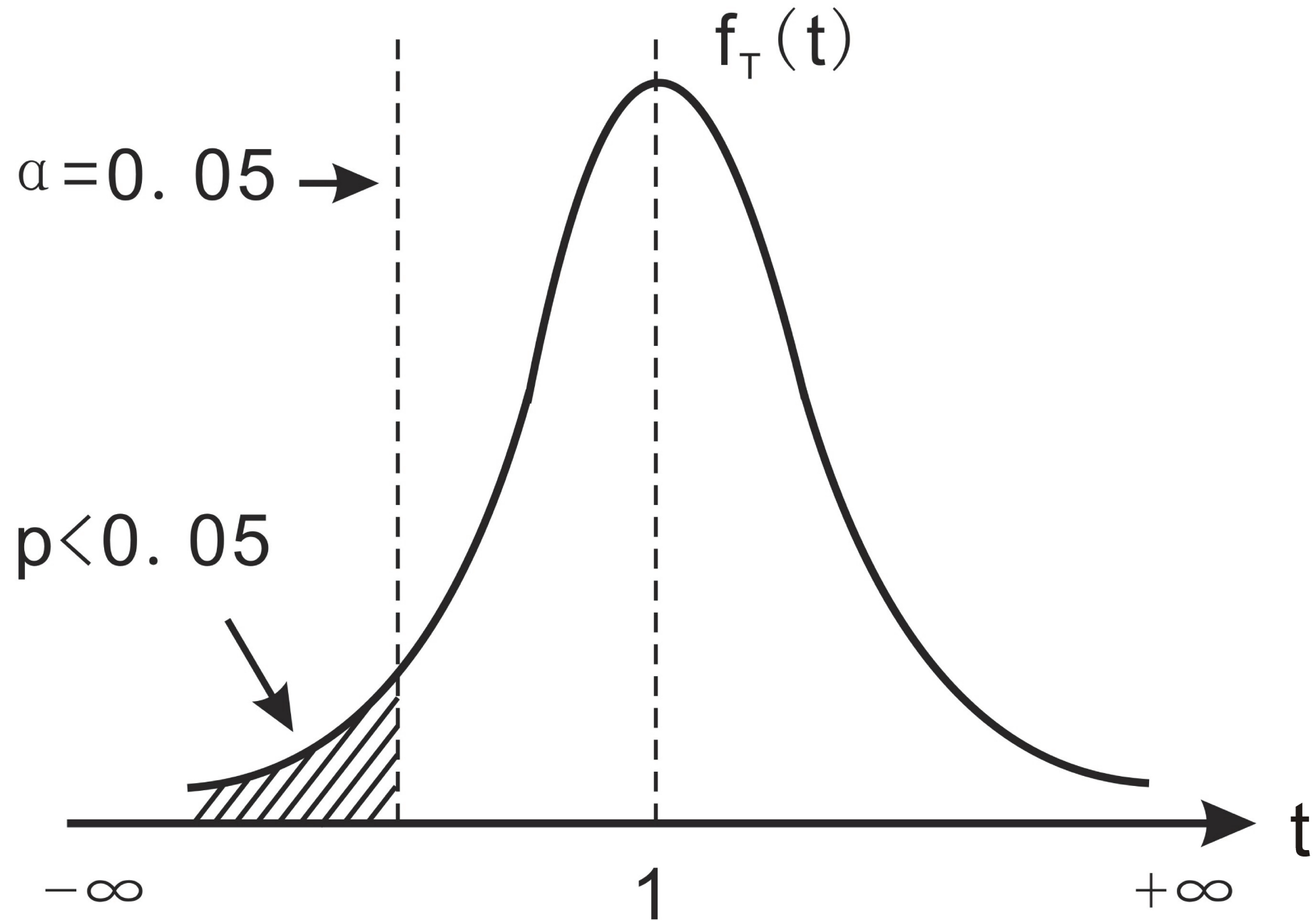

Supplement: Additional file 1: — Diagrammatic sketch of the analytical distribution function fT(t). Description: t represents the co-expression change of a miRNA — mRNA pair between earlier stage and later stage. If the clinical value t * locates in the shadow region (p < 0.05), the corresponding pair is regarded as having significant regulation change between the two stages. [file 12918_2015_145_MOESM1_ESM.pdf]
