# Supplementary material for: microRNAs’ differential regulations mediate the progress of Human Papillomavirus (HPV)-induced Cervical Intraepithelial Neoplasia (CIN)
Source: BMC Syst Biol. 2015 Feb 7;9:4. doi: 10.1186/s12918-015-0145-3 (PMC4337110; doi:10.1186/s12918-015-0145-3)
Supplement: Additional file 2: — Molecular elucidation of miRNA particular regulatory networks constructed solely based on literature review. This additional file provides detailed molecular elucidation for miRNA particular regulatory networks (Figures 6, 7, and 8) constituted by the 99 Efficient Pairs selected based on extensive literature review. Through our findings and inference, the potential molecular mechanisms for the course of CIN deterioration are provided from eight aspects: inflammation, angiogenesis, cellular stress, apoptosis & proliferation, invasion & migration, virus infection, cell immortalization, and energy metabolism. [file 12918_2015_145_MOESM2_ESM.pdf]

Molecular elucidation of miRNA particular regulatory networks constructed solely based on literature review

—— for systematically revealing mechanisms of CIN progress

This additional file provides detailed molecular elucidation for the 99 Efficient Pairs detected by the SIG++ algorithm, which constitute miRNAs' particular regulatory networks (Fig. 6, Fig. 7, and Fig. 8) for normal, CIN I, and CIN III stages, respectively, to systematically reveal the potential mechanisms for HPV-induced CIN progress. The 99 miRNA — mRNA pairs with significant regulation change include 78 miRNAs and 69 mRNAs. In the following, based on our findings, we provide potential molecular mechanisms for the course of CIN deterioration, from eight aspects: inflammation, angiogenesis, cellular stress, apoptosis & proliferation, invasion & migration, virus infection, cell immortalization, and energy metabolism. On the whole, we found that for the CIN progress, inflammation happened persistently, angiogenesis was enhanced, cell's ability to tackle stress was boosted, cells proliferated continuously, the capability for invasion and migration was enhanced, energy metabolism was strengthened, HPV replication and re-infection was augmented, and HPV's integration into host genome exhibited gradually increased prevalence. Especially, at CIN I stage, the NF-κB pathway and TGF-β pathway had the strongest activity, virus replication was most active, and the energy metabolism was most animated. At CIN III stage, the G1/S transition was initiated abundantly, and the cellular status of suffering from selection pressure emerged, therefore virus began to choose integration as the major existing way of staying in host cells. As follows, based on the detected Efficient Pairs, we detailedly in details our findings on the potential mechanisms of the three processes: HPV gradually integrated into cell genome during CIN progress, host cells gradually transformed into immortalization, and how virions as well as infected cells successfully escaped immune clearance when surrounded by the environment with intensive inflammation reactions.

Inflammation persistently happened during CIN progress

It was reported that during the CIN progress, inflammation persistently happened (Hammes, 2007). The inflammatory reactions inside the infected cells are manifested by the activation of NF-κB pathway as well as the TGF-β pathway, and outside the infected cells are majorly reflected by the recruitment of lymphocytes into the pathologic tissue, thus the above two aspects together contribute to inflammation persistence and increment. Our findings provide some clues for how those processes happened.

For the NF-κB pathway, miR-326, miR-766, and miR-93 together inhibited TNFRSF21 only in normal stage, but not in CIN I and CIN III stages; miR-455-3p inhibited ELF3 only in normal stage, but not in CIN I and CIN III stages; miR-584 did not regulate CARD8 at normal stage, but at CIN I and CIN III stages; miR-155 did not regulate DUSP14 at normal and CIN I stages, but at CIN III stage (Fig. 6 ~ Fig. 8). Thus TNFRSF21 and ELF3' expression increased at CIN I and CIN III stages, CARD8's expression decreased at CIN I and CIN III stages, DUSP14's expression decreased at CIN III stage (Fig. 6 ~ Fig. 8). TNFRSF21 encodes a member of TNF-α receptor family, which mediates TNF-α's activating on NF-κB (Kasof, 2001). The protein encoded by ELF3 can activate NF-κB pathway in prostate cancer (Longoni, 2013). The protein encoded by CARD8 contains a domain which can recruit caspase and activate it; meanwhile, this protein can repress the activation of NF-κB (Razmara, 2002). The protein encoded by DUSP14 can prevent NF-κB activity by dephosphorylating TAK1 (Zheng, 2013). The above findings suggest that TNFRSF21 and ELF3' high expression, and CARD8's low expression since CIN I stage, promoted the activation of NF-κB pathway since CIN I stage; moreover, DUSP14's decreased expression in CIN III stage further accelerated NF-κB's activity at that time.

For the TGF-β pathway, miR-423-5p's repression on THY1 did not exist in normal stage, but in CIN I and CIN III stages; miR-20b's repression on RASL11B existed in normal and CIN I stages, but not in CIN III stage (Fig. 6 ~ Fig. 8). Therefore, THY1's expression decreased in CIN I and CIN III stages, and RASL11B's expression increased in CIN III stage (Fig. 6 ~ Fig. 8). THY1 is also termed CD90, its encoded protein can

block the activation of TGF-β pathway in lung fibroblasts (Zhou, 2010). RASL11B is similar to RAS gene, and can mediate TGF-β pathway to promote inflammation and carcinogenesis (Stolle, 2007). Those suggest that THY1's decreased expression since CIN I can promote TGF-β pathway activation in CIN I and CIN III stages; in CIN III stage, RASL11B's upregulated expression may further promote activation of TGF-β pathway.

In addition, miR-32's suppression on SMAD7 did not exist in normal stage, but in CIN I and CIN III stages; miR-495 inhibited SMAD7 only in normal and CIN I stages, but not in CIN III stage (Fig. 6 ~ Fig. 8). Thus in CIN I stage, miR-32 and miR-495 both inhibited SMAD7, leading to SMAD7's decreased expression in CIN I (Fig. 6 ~ Fig. 8). It has been known that SMAD7 has regulatory effect both on NF-κB and TGF-β pathways. In hepatocarcinoma cells, SMAD7 can block NF-κB pathway by interacting with TAB2 (Wang, 2013), and can also repress TGF-β pathway by preventing SMAD3 phosphorylation (Wang, 2013). It suggests that in CIN I stage, the downregulation of SMAD7 expression may promote activation of both NF-κB and TGF-β pathways. To sum up, during the whole CIN progress, NF-κB pathway and TGF-β pathway were both continuously activated, thereby maintaining chronic inflammation response inside the infected cells.

On the other hand, in the extracellular microenvironment of infected cells, our results found that plenty of chemokines were highly expressed, which recruited lymphocytes to the cervical tissue, thereby boosting local inflammation reactions. miR-425 repressed MAP3K5 only in normal stage, but not in CIN I and CIN III stages; miR-181a, miR-181b, miR-181c, and miR-539 together inhibited CXCL1 in normal stage, and at CIN I and CIN III stages those regulations disappeared; miR-449b — CCL22's and miR-507 — CCL20's regulation only existed in normal and CIN I stages, but not in CIN III stage; miR-296-5p only repressed FLNB in normal stage, but not in CIN I and CIN III stages (Fig. 6 ~ Fig. 8). Therefore, the expression of MAP3K5, CXCL1, and FLNB increased in CIN I and CIN III stages, the expression of CCL22 and CCL20 were upregulated in CIN III stage (Fig. 6 ~ Fig. 8). MAP3K5 is a member of MAPK signaling pathway, and can enhance the production of proinflammatory mediators

(Mnich, 2010). CXCL1 encodes a chemokine, which plays important role in inflammation via recruiting neutrophilic granulocyte (Ritzman, 2010), leading to the phenomenon that the number of neutrophilic granulocyte was on the increase during CIN progress (Bais, 2005). The chemokine encoded by CCL22 can recruit T cells, and the chemokine encoded by CCL20 can recruit Th17 cells. FLNB encodes the actin filament, which can drive leukocyte transendothelial migration (Kanters, 2008). The above findings suggest that at CIN I and CIN III stages, MAP3K5's upregulated expression induced proinflammatory mediator production, CXCL1's high expression promoted neutrophilic granulocyte recruitment; at CIN III stage, CCL22 and CCL20' increased high expression promoted the recruitment of T cells and Th17 cells, respectively; while during the whole CIN progress (CIN I and CIN III stages), FLNB's high expression facilitated leukocyte transendothelial migration into the tissue. Therefore, as CIN advanced, lymphocytes continuously assembled into the cervical tissue, thus causing the chronic inflammation microenvironment formation and intensification.

Based on the above findings, chronic inflammation persistently happened in the CIN progress. However, how virus and infected cells can still escape immune clearance successfully? The miRNA — mRNA pairs with significant regulation change detected in this study may provide some clues for this process.

miR-372 inhibited MASP1 expression in CIN I and CIN III stages, but not in normal stage, thus MASP1 was downregulated since CIN I (Fig. 6 ~ Fig. 8). MASP1 encodes serine protease, which can activate lectin complement pathway and promote complement response (Sekine, 2013). The activated complement bonds to the extracellular virus episome and induce its neutralization and lysis (Alcami, 2000). Because of MASP1's downregulation as well as the probably inhibited complement response in CIN I and CIN III stages, HPV virions can infect new cells in that period.

miR-660 inhibited APLP2 in normal stage and the regulation disappeared in CIN I and CIN III stages (Fig. 6 ~ Fig. 8). APLP2 encodes amyloid precursor-like protein, which promotes the endocytosis of MHC-I on cell surface (Peters, 2011). MHC-I in lower effective concentration can weaken Tc cell activity in cell-mediated immunity. It

was known that the Tc cell-mediated immunity targeting HPV E6 and E7 can induce CIN regression (Sarkar, 2005). Therefore, our result shows one possible reason why the infected cells can escape Tc cell cytotoxicity.

In normal stage, miR-29a, miR-29b, miR-30b, miR-30c, miR-30d, miR-452, miR-582-5p, and miR-627 inhibited CLEC2D, miR-485-5p inhibited CEACAM1, but in CIN I and CIN III stages, those regulation all disappeared, and CLEC2D and CEACAM1' expression was highly increased (Fig. 6 ~ Fig. 8). The protein encoded by CLEC2D is a surface protein of the infected cells, which can interact with Natural killer cell (NK cell)'s surface protein CD161 and thus inhibit the cytotoxicity from NK cell (Aldemir, 2005). CEACAM1 encodes a carcinoembryonic antigen, and can repress the expression of NK cell ligand NKG2D on tumor cell surface (Chen, 2011), thus preventing NK cell cytotoxicity.

Given that CIN can be effectively dissipated by cell-mediated immunity (Sarkar, 2005), based on the above, once again we underline the possible process of infected cells escaping from immune clearance. NK cell and Tc cell can kill and clean out the infected cells by cytotoxicity. In CIN I and CIN III stages, endocytosis for MHC-I was enhanced inside the infected cell, thus preventing MHC-I molecular-mediated E6 and E7 antigen presentation in order to avoid Tc cell activation. However, deletion of MHC-I on infected cell surface makes the infected cell susceptible to be attacked by NK cells, since MHC-I molecule is the signal for negatively regulating NK cell activity (Textor, 2008). We found in CIN I and CIN III stages, infected cells not only prevented MHC-I molecular, but also prevented the NK cell activity by CD161 and NKG2D pathways. Therefore, infected cell escaped cellular immunity and survived.

Angiogenesis was gradually enhanced during CIN progress

The intensive formation of blood vessels entangled in tissue is an important microenvironment character for the mass of local cells transforming into tumor. The regulation of miR-455-3p — ELF3, miR-425 — MAP3K5, only existed in normal stage, but not in CIN I and CIN III stages; miR-939's repression on SPARC, not existed in normal stage, but in CIN I and CIN III stages; miR-548b-5p only inhibited SOX18 at

normal and CIN I stages, but not at CIN III stage (Fig. 6 ~ Fig. 8). Thus since CIN I stage, the expression of ELF3 and MAP3K5 increased, and SPARC's expression decreased; at CIN III stage, SOX18's expression was upregulated (Fig. 6 ~ Fig. 8). The protein encoded by ELF3 can promote the expression of Angiopoietin-1 under inflammation surroundings, thereby inducing angiogenesis (Brown, 2004). MAP3K5 can activate the expression of VEGF by mediating IL-6 signaling, resulting in robust angiogenesis (Tzeng, 2013). SPARC encodes a cysteine-rich acidic matrix-associated protein, which can downregulate VEGF expression in gastric cancer to block angiogenesis (Zhang, 2012). SOX18 has a role in cervical cancer by facilitating angiogenesis (Petrovic, 2009). The above findings suggest that the upregulated expression of ELF3 and MAP3K5 as well as the downregulated expression of SPARC since CIN I stage, may promote angiogenesis process in cervical tissue; at CIN III stage, SOX18's increased expression may further advance angiogenesis.

Besides, it is interesting that miR-137 and miR-376a together only inhibited SULF1 in normal and CIN I stages, but not in CIN III stage, thus SULF1's expression increased at that time (Fig. 6 ~ Fig. 8). SULF1 encodes an extracellular heparan sulfate endosulfatase, which selectively removes 6-O-sulfate groups from heparan sulfate chains of heparan sulfate proteoglycans, and has an important antitumor function in ovarian cancer through its antiangiogenic role (Liu P, 2012). Hence, it suggests that SULF1's increased expression in CIN III stage may suppress angiogenesis, therefore contributing to the survival selection pressure at CIN III stage to some extent.

To sum up, the process of angiogenesis during the CIN development is that blood vessels emerged inevitably as CIN progressed, therefore creating the environment appropriate for tissue evolving into tumor.

Infected cells' ability for withstanding cellular stresses was gradually improved during CIN progress

When cells are in the abnormal status, they are faced with intracellular stresses, majorly including endoplasmic reticulum stress (ER stress) and oxidative stress, which directly cause damage to cells.

The ER stress means a condition with disturbance in any of endoplasmic reticulum functions, resulting in the disruption of the proper folding and secretory capacity of the ER and increased load of unfolded proteins. If the ER stress continuously happen, cell's physiological function will be destroyed and the cell finally work to its death. The regulation of miR-576-5p — AGR2, miR-9 — ERP27, only existed in normal stage, but not in CIN I and CIN III stages; miR-939' repression on SPARC did not exist in normal stage, but in CIN I and CIN III stages (Fig. 6 ~ Fig. 8). Thus since CIN I stage, the expression of AGR2 and ERP27 was increased, and the expression of SPARC was decreased (Fig. 6 ~ Fig. 8). AGR2 can arrest the ER stress-induced apoptosis (Ryu, 2013). ERP27 encodes an endoplasmic reticulum protein, which can alleviate the ER stress (Alanen, 2006). The protein encoded by SPARC can induce endoplasmic reticulum stress, leading to autophagy-mediated apoptosis in neuroblastoma (Sailaja, 2013). Those suggest that since CIN I stage, AGR2 and ERP27 were significantly upregulated, and SPARC was downregulated, probably resulting in relieving ER stress and promoting cell survival during the ER-stressed environment.

On the other hand, in the inflammation surroundings, cells are often attacked by the intracellular superoxide radicals, which impose oxidative stress, causing damage to the organelles. The regulation of miR-425 — MAP3K5, miR-629 — MGST1, only existed in normal stage, but not in CIN I and CIN III stages; miR-203's suppression on BCHE existed in normal and CIN I stages, but not in CIN III stage (Fig. 6 ~ Fig. 8). Therefore, since CIN I stage, MAP3K5 and MGST1' expression increased; at CIN III stage, BCHE's expression was upregulated (Fig. 6 ~ Fig. 8). MAP3K5 participates in numerous stress responses, including oxidative stress (Shiizaki, 2013). The protein encoded by MGST1 is predominantly located in the endoplasmic reticulum and outer mitochondrial membrane, and has significant protection against agents that are known to induce lipid peroxidation (Johansson, 2010). Mitochondria in MGST1-overexpressing cells were shown to be protected from oxidative insult as measured by calcium loading capacity and respiration (Johansson, 2010). BCHE encodes the butyrylcholinesterase, which has antioxidant activity in pregnant women

(Omu, 2010). The above suggest that MAP3K5 and MGST1' upregulated expression since CIN I stage, and BCHE's increased expression at CIN III stage, may promote cells' response to oxidative stress, and protect organelle membranes, to diminish oxidative damage.

To sum up, since CIN I stage, cells' ability for resisting ER stress and oxidative stress was enhanced, and at CIN III stage, cells' capacity for withstanding stress was further strengthened.

Apoptosis was arrested and cells proliferated continuously during CIN progress

Along the course of CIN progress, cells successfully survived, and their proliferation potency was greatly enhanced. Our results provide a number of clues for revealing the intracellular intricate network happened for guiding cell survival and proliferation during CIN progress.

For the apoptosis, miR-300's repression on FILIP1L, did not exist in normal stage, but in CIN I and CIN III stages (Fig. 6 ~ Fig. 8). Thus FILIP1L's expression was downregulated since CIN I (Fig. 6 ~ Fig. 8). FILIP1L encodes a filamin A interacting protein, whose overexpression can inhibit cell growth and promote apoptosis in ovarian cancer (Kwon, 2008). It suggests that FILIP1L's decreased expression since CIN I stage may inhibit cell apoptosis. miR-32's suppression on SMAD7 did not exist in normal stage, but in CIN I and CIN III stages; miR-495 inhibited SMAD7 only in normal and CIN I stages, but not in CIN III stage (Fig. 6 ~ Fig. 8). Thus at CIN I stage, both miR-32 and miR-495 repressed SMAD7, and caused SMAD7's decreased expression in CIN I stage (Fig. 6 ~ Fig. 8). SMAD7 can promote apoptosis in hepatoma carcinoma cells, by mediating caspase9-caspase3 pathway (Wang, 2013). It suggests that in CIN I stage, SMAD7's downregulated expression may block cell apoptosis. On the other hand, miR-137 and miR-376a' repression on SULF1 existed in normal and CIN I stages, but not in CIN III stage (Fig. 6 ~ Fig. 8). Thus SULF1 expression increased in CIN III stage. SULF1 has remarkable antitumor role in ovarian cancer by inducing apoptosis (Liu P, 2012). It suggests that SULF1's upregulated expression in CIN III stage may make cells tend to apoptosis, thereby imposing survival selection pressure at CIN III

stage. To summarize, our results found that apoptosis was repressed since CIN I stage, especially blocked to the greatest degree in CIN I stage; while in CIN III stage, cells were vulnerable to apoptosis, forming the selection pressure.

For the cell cycle and proliferation, miR-423-5p's suppression on THY1 did not exist in normal stage, but in CIN I and CIN III stages (Fig. 6 ~ Fig. 8). Thus from CIN I stage, THY1's expression was downregulated (Fig. 6 ~ Fig. 8). THY1 functionally works as a tumor suppressor gene in nasopharyngeal carcinoma by blocking cell cycle G0/G1 transition (Lung, 2010). It suggests that THY1's decreased expression since CIN I stage may facilitate G0/G1 transition, thereby inducing cell proliferation.

miR-939's regulation on SPARC, miR-129-5p, miR-377, miR-380, and miR-661' regulation on ADARB1, did not exist in normal stage, but in CIN I and CIN III stages; the regulations of miR-181b on CDKN3, miR-548d-5p on DDIT4, miR-497 on CCNE1, existed in normal stage, but not in CIN I and CIN III stages; the regulations of miR-215 on CDC7, miR-216b on STK38L, miR-940 on SH2B3, existed in normal and CIN I stages, but not in CIN III stage (Fig. 6 ~ Fig. 8). Thus since CIN I, the expression of SPARC and ADARB1 was downregulated, while the expression of CDKN3, DDIT4, and CCNE1 was upregulated; at CIN III stage, CDC7, STK38L, and SH2B3' expression increased (Fig. 6 ~ Fig. 8). SPARC overexpression in neuroblastoma can activate tumor suppressor protein PTEN to block cell cycle G1/S transition, thereby inhibiting cell proliferation (Bhoopathi, 2012). ADARB1 encodes the enzyme responsible for pre-mRNA editing of the glutamate receptor subunit B by site-specific deamination of adenosines, and the encoded protein can promote p27 and p21 protein expression, resulting in G1/S transition restraint (Galeano, 2013). The protein encoded by CDKN3 belongs to the dual specificity protein phosphatase family, and is overexpressed in cervical cancer as a marker for CIN malignancy degree (Espinosa, 2013). Moreover, in hepatoma carcinoma cells, CDKN3 can facilitate G1/S transition to promote cell proliferation (Xing, 2012). DDIT4 overexpression can activate Akt, thus promoting G1/S transition (Jin, 2013). CCNE1 encodes cyclin E1, which allows G1/S passage. CDC7 can promote G1/S transition. STK38L encodes a serine/threonine kinase, which facilitates G1/S transition by regulating p21 stability (Cornils, 2011). SH2B3 can activate Akt pathway,

thereby promoting G1/S transition (Fitau, 2006). Those suggest that since CIN I stage, SPARC and ADARB1' decreased expression, CDKN3, DDIT4, and CCNE1' increased expression, may promote G1/S transition, thus facilitating cell proliferation; at CIN III stage, CDC7, STK38L, and SH2B3' upregulated expression may further promote G1/S transition to accelerate cell growth.

The regulation of miR-206 on KIF2A, miR-452 on KIF20A, miR-425 on TACC3, existed in normal stage, but not in CIN I and CIN III stages; the regulation of miR-323-3p on OIP5, miR-376a, miR-522, and miR-548c-5p on C14orf106, miR-641 on PRC1, existed in normal and CIN I stages, but not in CIN III stage (Fig. 6 ~ Fig. 8). Thus since CIN I stage, KIF2A, KIF20A, and TACC3' expression increased; at CIN III stage, OIP5, C14orf106, and PRC1 were overexpressed (Fig. 6 ~ Fig. 8). The protein encoded by KIF2A is a plus end-directed motor required for normal mitotic progression, through maintaining normal spindle activity and promoting chromosome movement (Manning, 2007). The protein encoded by KIF20A functions in cytokinesis (Hill, 2000). TACC3 encodes a motor spindle protein that may play a role in stabilization of the mitotic spindle (Thakur, 2013). OIP5 and C14orf106 work as regulating centromere/kinetochore structure and function, in order to carry out cell cycle M phase (Fujita, 2007). PRC1 has an important role in cytokinesis by mediating G2/M transition (Liu, 2009). Those suggest that since CIN I stage, KIF2A, KIF20A, and TACC3' upregulated expression may promote cell cycle M phase progress; at CIN III stage, OIP5, C14orf106, and PRC1' increased expression may further boost M phase progress, thus further facilitate cell accomplishing proliferation.

To sum up, at CIN I stage, cell apoptosis was blocked to the greatest degree, thus cells have the maximum probability to survive in CIN I stage; since the CIN I stage, cell cycle was gradually activated, and cells' proliferation ability achieved its peak in CIN III stage.

Infected cells' invasion and migration ability was significantly enhanced during CIN progress

Along CIN development, the infected cells' ability for invasion and migration was

gradually enhanced, preparing for the large-scale aggressive invasion in the ultimate cervical cancer stage. Our results provide multiple potential molecular mechanisms for revealing the complicated intracellular network for the process of cell invasive capability increment.

The repression of miR-300 on FILIP1L, miR-423-5p on THY1, and miR-200a on CSTA, did not exist in normal stage, but in CIN I and CIN III stages; the regulation of miR-576-5p on AGR2, miR-206 on RHPN2, miR-203 on GALNT4, miR-665 on S100A2, miR-941 on VCAM1, and miR-485-5p on CEACAM1, only existed in normal stage, but not in CIN I and CIN III stages; the regulation of miR-641 on SCGB2A2, miR-632 on MYH11, only existed in CIN I stage, but not in normal and CIN III stages; the regulation of miR-204 and miR-548b-5p on HLTF, miR-519b-3p on NELL2, existed in normal and CIN I stages, but not in CIN III stage (Fig. 6 ~ Fig. 8). Thus from the CIN I stage, FILIP1L, THY1, and CSTA' expression was downregulated, AGR2, RHPN2, GALNT4, S100A2, VCAM1, and CEACAM1' expression was upregulated, SCGB2A2 and MYH11' expression only decreased in CIN I stage, HLTF and NELL2' expression increased at CIN III stage (Fig. 6 ~ Fig. 8). FILIP1L's downregulated expression in ovarian cancer can promote cell invasion (Burton, 2011). THY1's downregulation in ovarian cancer can promote cell proliferation and migration (Yang, 2009). AGR2 has an important oncogenic role by facilitating cell migration (Chevet, 2013). SCGB2A2 inhibits cell invasion and migration in breast cancer (Koh, 2014). RHPN2 encodes a Ras-homologous (Rho)-GTPase binding protein, which is involved in the organization of actin cytoskeleton by repressing stress fiber formation (Peck, 2002). Stress fibers can generate membrane tension, which induces integrin assemble, thereby recruiting other components required for tight junction (Chrzanowska-Wodnicka, 1996). Therefore, RHPN2-induced inhibition of stress fiber formation may eliminate intercellular tight junction to promote cell invasion. It is also has been known that RHPN2 protein is expressed in Hela cells, thus there is little actin stress fiber in the cells (Peck, 2002). The protein encoded by MYH11 is a smooth muscle myosin belonging to the myosin heavy chain family, functions as a major contractile protein, and can depolymerize the intracellular stress fiber (Tanaka, 1998). The protein

encoded by CSTA is expressed on the cytomembrane of keratinocyte, and functions essentially in the adhesion between keratinocytes; if this gene is depleted, the cell adhesion will be dissolved (Blaydon, 2011). GALNT4 encodes a member of the UDP-N-acetyl-alpha- D-galactosamine:polypeptide N-acetylgalactosaminyltransferase (GalNAc-T) family of enzymes, which can make the target protein O-glycosylation (Wandall, 2007). While O-glycosylation determines and promotes tumor malignancy, via facilitating cell invasion and migration (Ono, 2004). S100A2 encodes an EF hand calcium-binding protein, which can be upregulated by TGF-β, and promote cell invasion and migration (Neesse, 2007). The protein encoded by HLTF has helicase and ATPase activities, and can modify chromosome structure. HLTF is activated in the initial steps of carcinogenesis to promote tumor formation, and its overexpression in Hela cells can enhance cell migration by inducing PAI-1 expression (Debauve, 2008). NELL2 can promote cell invasion (Kim, 2013). VCAM1 encodes vascular cell adhesion molecule, which is induced by EGF and can promote tumor cell invasion (Zheng, 2013). CEACAM1 encodes a member of the carcinoembryonic antigen (CEA) gene family, which can inhibit the junction between cell and extracellular matrix, thus facilitating cell invasion (Liu, 2013). The above suggest that since CIN I stage, FILIP1L, THY1, and CSTA' decreased expression, and AGR2, RHPN2, GALNT4, S100A2, VCAM1, and CEACAM1' increased expression, may enhance cells' capacity of invasion and migration; SCGB2A2 and MYH11' downregulated expression in CIN I stage, as well as HLTF and NELL2' upregulated expression in CIN III stage, may specially promote cell invasion at CIN I and CIN III, respectively. To sum up, during the whole CIN progress, cells' potency for invasion and migration was greatly improved.

Prevalent HPV integration only happened since CIN III stage

In cervical cancers, the presence of HPV in cells is entirely in the form of integration (Woodman, 2007). In stage CIN I and CIN II, infected cells contained HPV mainly in episomal form; while in stage CIN III, these were superseded by cells carrying only integrated virus (Ruutu, 2002). To our acknowledge, so far there is little molecular mechanism report on why HPV integration only happened since CIN III

stage.

Comparing Fig. 6 ~ Fig. 8 for the course of miRNA — mRNA regulation change, we found that miR-125a-3p repressed OAS2 in normal and CIN I stages, and this regulation disappeared in CIN III stage, resulting in significant increment of OAS2 expression in CIN III stage. OAS2 encodes the 2'-5' oligoadenylate synthetase, which can degrade virus RNA and inhibit virus replication (Silverman, 2007). It suggests that at CIN III stage, the intracellular environment exerted selection pressure on HPV, which made virus episome's replication and transcription very difficult to happen. On the other hand, after integration, HPV genes can be persistently co-replicated with host genome; at the same time, HPV E6 and E7 genes' transcription was no longer repressed by E2; furthermore, the virus transcript from integrated host genome was more stable than the transcript from episome (Woodman, 2007). Therefore, at CIN III stage, HPV's strategy of integrating itself into host cell genome has much greater growth advantage.

miR-137 and miR-376a repressed SULF1's expression at normal and CIN I stages, while at CIN III stage those regulation disappeared, resulting in significant increment of SULF1 expression (Fig. 6 ~ Fig. 8). SULF1 encodes an extracellular heparan sulfate endosulfatase, which can remove 6-O-sulfate groups from heparan sulfate chains of heparan sulfate proteoglycans. This gene has remarkable anti-tumor function in ovarian cancer (Liu, 2012), such as preventing angiogenesis, arresting cell proliferation, and promoting cell apoptosis. It suggests that at CIN III stage, SULF1's high expression may impose pressure for cell survival. While after integration, E6 and E7 proteins' abundant expression can prevent cell apoptosis and promote cell proliferation. Therefore, at CIN III stage, the integration of HPV has preferential advantage of cell survival.

miR-129-5p and miR-938 did not regulate PRDM1 at normal stage, but at CIN I and CIN III stages, thus PRDM1's expression was decreased (Fig. 6 ~ Fig. 8). PRDM1 encodes a protein that acts as a repressor of interferon-β (IFN-β) gene expression, by binding specifically to IFN-β gene promoter (Keller, 1991) It was reported that

IFN-β treatment of cervical keratinocytes, which were naturally infected with HPV16 episomes, can promote rapid reduction in HPV episome numbers and enhance prevalent emergence of HPV integration (Herdman, 2006). It suggests that IFN-β has the ability of clearing up HPV infection, at the same time it also provides the selection pressure for integration. Therefore, in CIN I and CIN III stages, PRDM1 was down-regulated, and may induce IFN-β production as well as the consequent HPV integration.

Furthermore, miR-654-3p's repression on FBXO5 only disappeared at CIN III stage, therefore FBXO5's expression was increased in CIN III stage (Fig. 6 ~ Fig. 8). miR-106a, miR-17, and miR-23a' repression on SC4MOL only existed in normal stage, while did not exist at CIN I and CIN III stages, resulting in the increment of SC4MOL expression (Fig. 6 ~ Fig. 8). The protein encoded by FBXO5 is a subunit of protein ligase complex, and its high expression can promote genomic instability (Yu, 2013). SC4MOL encodes a methyl sterol oxidase, which catalyzes demethylation of C4-methylsterols (the meiosis-activating sterols) in the cholesterol synthesis pathway (He, 2011). Therefore, SC4MOL encodes a meiosis-specific protein. It was reported that the atypical expression of meiosis-specific proteins in high-risk HPV-associated tumor could induce genomic instability (Duensing, 2004). The above findings suggest that SC4MOL's high expression in CIN I and CIN III stages and FBXO5's high expression in CIN III stage may promote genomic instability to facilitate HPV integration.

On the other hand, based on the Efficient Pairs, we also analyzed the potential molecular mechanisms of HPV replication and re-infection during CIN progress. The pairs of miR-92a — SLC11A2, miR-206 — WEE1, miR-17 — TBC1D2, and miR-93 — TBC1D2 had regulation at normal stage, and those regulation disappeared in CIN I and CIN III stages, resulting in the increased expression of SLC11A2, WEE1, and TBC1D2 (Fig. 6 ~ Fig. 8). SLC11A2 encodes a solute carrier protein and is involved in iron absorption, while iron is essential for host cell synthesis of virions (Simonart, 2002). WEE1 encodes a G2 checkpoint kinase, and its increased expression induces S phase reentry to facilitate HPV DNA replication in human keratinocytes (Banerjee, 2011). TBC1D2 encodes the key protein for cellular endocytic trafficking (Serva, 2012),

while HPVs used the endocytosis pathway to enter cells leading to repeated infection (Bousarghin, 2003). The above analysis suggest that based on miRNA — mRNA differential regulation, the virion replication and re-infection were intensified during CIN progress.

To sum up, our findings in this section focused on exploring the process of how intracellular selection pressure gradually happened to compel HPV integration, i.e. at CIN I stage, HPV virions were intensively replicated in keratinocytes and repeatedly infected new host cells; and at CIN III stage, selection pressure was the major cellular environment, thus the infected cells finally survived in the way of HPV integration. The above description of regulatory process may provide molecular foundation for the clinical discovery that cervical cancer has developed from a single dysplastic cell clone (Vinokurova, 2005).

Keratinocytes gradually transformed into the immortal cells during CIN progress

During the development of HPV-induced CIN, host cells gradually became immortalized malignant cells. Our findings provide some molecular clues for this process.

miR-106b and miR-17' repression on KPNA2, miR-181a,miR-181b, and miR-181c' repression on TIMELESS only existed in normal stage but not in CIN I and CIN III stages, thereby the expression of KPNA2 and TIMELESS were increased (Fig. 6 ~ Fig. 8). The protein encoded by KPNA2 is involved in nuclear import of proteins, and can help HPV E6 protein transporting into nucleus (Le Roux, 2003), then E6 can bind to nuclear MYC and induce the expression of telomerase reverse transcriptase (TERT), which is the catalytic subunit of telomerase (Oh, 2001). On the other hand, the protein encoded by TIMELESS preserves telomere length by promoting efficient DNA replication through human telomeres (Leman, 2012). Those suggest that at CIN I and CIN III stages, the expression of telomerase was enhanced, and telomere length was maintained, thus promoting immortalization of infected cells.

TFCP2L1 was repressed by miR-15b only in normal stage, and this regulation disappeared in CIN I and CIN III stages, resulting in increased expression of TFCP2L1

(Fig. 6 ~ Fig. 8). TFCP2L1 encodes a transcription factor, which participates in maintaining pluripotency in embryonic stem cells (To, 2010). It suggests that the increment of TFCP2L1 expression at CIN I and CIN III stages may promote the infected cells transforming into pluripotency.

At normal and CIN I stages, miR-299-5p and miR-632 repressed PDZD2, miR-105 and miR-548a-3p repressed GPC4, while those regulation disappeared in CIN III stage, thus PDZD2 and GPC4‘ expression increased (Fig. 6 ~ Fig. 8). PDZD2 encodes a PDZ domain containing protein, and is highly expressed in infant cells, facilitates cell proliferation and inhibits differentiation (Suen, 2008). GPC4 encodes the glypican, which is specifically required to maintain the self-renewal, pluripotency, and tumorigenic potential of embryonic stem cells (Fico, 2012). Therefore, the higher expression of PDZD2 and GPC4 at CIN III stage may further boost pluripotency in the infected cells.

At normal and CIN I stages, miR-522 and miR-920 repressed DVL3, and miR-622 repressed AXIN2, while at CIN III stage, those regulation disappeared and the expression of DVL3 and AXIN2 were elevated (Fig. 6 ~ Fig. 8). DVL3 encodes a cytoplasmic phosphoprotein which can activate the Wnt/β-catenin signaling in cervical cancer (Kwan, 2013). AXIN2 encodes the Axin-related protein, which plays an important role in the regulation of the stability of beta-catenin in the Wnt signaling, and can promote cell proliferation in ovarian cancer (Schmid, 2011). The above findings suggest that at CIN III stage, deregulations happened in Wnt signaling thus contributing to the genesis of malignancy.

To sum up, based on the above analysis, as early as in CIN I stage, infected cells began to exhibit the characteristics of immortalization such as inducing telomerase expression and preserving telomere length; then in CIN III stage, the Wnt signaling was activated, and cells’ potential for pluripotency was enhanced, therefore the infected cells gained further advancement to immortalization.

Cells’ energy metabolism was overall animated during CIN progress, especially exhibiting glycolysis feature

Along the CIN progress, there must be significant abnormalities happened in cells' energy metabolism; however, currently there is little reports on this black box process. Our results of miRNA — mRNA differential regulations provide several clues for the energy metabolism features during CIN progress.

For the Krebs cycle pathway, miR-582-5p's repression on ACO2 existed in normal stage, but not in CIN I and CIN III stages (Fig. 6 ~ Fig. 8). Thus since CIN I stage, ACO2 expression was obviously upregulated (Fig. 6 ~ Fig. 8). ACO2 encodes an enzyme that catalyzes the interconversion of citrate to isocitrate via cis-aconitate in the second step of the TCA cycle. It has been known that the TCA cycle can promote tumor cell growth and proliferation (DeBerardinis, 2008). This suggests that ACO's increased expression since CIN I stage, may accelerate the TCA cycle, thereby providing sufficient fuels for cells.

For the Na+-K+- pump, the regulation of miR-224 on ATP1B3, and miR-342-3p on FXYD3, only existed in normal stage, but not in CIN I and CIN III stages (Fig. 6 ~ Fig. 8). Thus since CIN I stage, ATP1B3 and FXYD3 were overexpressed (Fig. 6 ~ Fig. 8). The proteins encoded by ATP1B3 and FXYD3 both belong to the Na+/K+ -ATPase family, which work against the ion gradients to pump 3 intracellular Na+ outwards the cell, and pump 2 extracellular K+ inwards the cell, by consuming one ATP. On the other hand, the potential energy released by Na+'s movement along the ion gradient can generate ATP. The Na+-K+- pump proteins are highly expressed in cervical diseases, and have already worked as the marker for clinical diagnosis and therapy (Grogan, 2010). And the significant ion gradients between two sides of cytomembrane formed by Na+-K+- pump, store biological energy which can be employed by cells (Brown, 1983). Furthermore, Na+-K+- pump can activate the glycolysis pathway (Pellerin, 1996). Those suggest that ATP1B3 and FXYD3' increased expression since CIN I stage, may promote Na+-K+- pump activity to enhance cell energy and activate the glycolysis pathway.

For the glucose metabolism, the regulation of miR-135a and miR-181c on SORD, miR-30b, miR-30c, and miR-30d on GM2A, only existed in normal stage, but not in CIN I and CIN III stages (Fig. 6 ~ Fig. 8). Thus from CIN I stage, SORD and GM2A'

expression were increased (Fig. 6 ~ Fig. 8). SORD encodes sorbitol dehydrogenase, which participates in glucose metabolism, by oxidizing the sorbitol to fructose using NAD(+) cofactor. GM2A encodes a small glycolipid transport protein which acts as a substrate specific co-factor for the lysosomal enzyme beta-hexosaminidase A. GM2A promotes the glucose metabolism in cells (Higashi, 2011). It was reported that tumor cells absorbed glucoses in a high rate with subsequent animated glusoce metabolism; and in cervical cancer, the activities of enzymes involved in glucose mechanism were significantly enhanced (MARSHALL, 1978). In the Hela cells, if glucose mechanism is inhibited, it will promote apoptosis induced by death receptor (Munoz-Pinedo, 2003). Those suggest that SORD and GM2A's upregulated expression since CIN I stage, may boost the glucose metabolism during CIN development.

For the lipid metabolism, miR-140-3p's suppression on PPAP2B, did not exist in normal stage, but in CIN I and CIN III stages (Fig. 6 ~ Fig. 8). Thus since CIN I stage, PPAP2B was significantly downregulated (Fig. 6 ~ Fig. 8). The protein encoded by PPAP2B is a membrane glycoprotein localized at the cell plasma membrane, and can actively hydrolyze extracellular lysophosphatidic acid and short-chain phosphatidic acid. It was reported that for the metabolism, fatty acid and glucose competed each other to be the substrate of respiration (Randle, 1998), i.e. glucose metabolism and lipid metabolism repress each other. It suggests that PPAP2B's decreased expression since CIN I stage, may inhibit cell lipid metabolism while promote glucose metabolism.

For the amino acid metabolism, the regulation of miR-199a-5p on SLC7A5, and miR-361-5p on MTRR, existed in normal stage, but not in CIN I and CIN III stages (Fig. 6 ~ Fig. 8). Thus since CIN I stage, SLC7A5 and MTRR were overexpressed (Fig. 6 ~ Fig. 8). SLC7A5 encodes a transporter for amino acid, which works in the intake of large neutral amino acid (Patel, 2013), and is highly expressed in cervical cancer (Uno, 2011). The protein encoded by MTRR, regenerates a functional methionine synthase via reductive methylation. Methionine is an essential amino acid required for protein synthesis and tumor growth (Hoffman, 1985). It suggests that since CIN I stage, SLC7A5 and MTRR' significantly increased expression may boost cells' intake of

amino acid, and the methionine metabolism may be strengthened, thus promoting cell growth.

To sum up, during CIN development, cell energy metabolism was majorly animated, and cells preferred to use glycolysis pathway.

To generalize, during HPV affected CIN progress, the infected cells promoted malignant development by multiple cooperative miRNA-mRNA regulatory changes, which induced immune escape, viral integration, cell proliferation and immortalization, angiogenesis, invasion and migration, etc., eventually leading to cancer formation.

References:

Alanen HI, Williamson RA, Howard MJ, Hatahet FS, Salo KE, Kauppila A, Kellokumpu S, Ruddock LW., ERp27, a new non-catalytic endoplasmic reticulum-located human protein disulfide isomerase family member, interacts with ERp57. *J Biol Chem*., 2006, 281(44):33727-33738.

Alcami A and Koszinowski UH, Viral mechanisms of immune evasion, *Immunology Today Review*, 2000, 21(9):447-455.

Aldemir H, Prod'homme V, Dumaurier MJ, Retiere C, Poupon G, Cazareth J, Bihl F, Braud VM., Cutting edge: lectin-like transcript 1 is a ligand for the CD161 receptor. *J Immunol.*, 2005, 175(12):7791-7795.

Bais AG and Beckmann I, A shift to a peripheral Th2-type cytokine pattern during the carcinogenesis of cervical cancer becomes manifest in CIN III lesions, *Journal of Clinical Pathology*, 2005, 58:1096-1100.

Banerjee NS, Wang HK, Broker TR, Chow LT, Human papillomavirus (HPV) E7 induces prolonged G2 following S phase reentry in differentiated human keratinocytes, *J Biol Chem*., 2011, 286(17):15473–15482.

Bhoopathi P, Gorantla B, Sailaja GS, Gondi CS, Gujrati M, Klopfenstein JD, Rao JS., SPARC overexpression inhibits cell proliferation in neuroblastoma and is partly mediated by tumor suppressor protein PTEN and AKT, *PLoS One*, 2012;7(5):e36093.

Blaydon DC, Nitoiu D, Eckl KM, Cabral RM, Bland P, Hausser I, van Heel DA, Rajpopat S, Fischer J, Oji V, Zvulunov A, Traupe H, Hennies HC, Kelsell DP, Mutations in CSTA, encoding Cystatin A, underlie exfoliative ichthyosis and reveal a role for this protease inhibitor in cell-cell adhesion, *Am J Hum Genet.*, 2011, 89(4):564-571.

Bousarghin L, Touze A, Sizaret PY, Coursaget P, Human papillomavirus types 16, 31, and 58 use different endocytosis pathways to enter cells, J Virology, 2003, 77(6):3846–3850.

Brown II, Galperin MY, Glagolev AN, Skulachev VP, Utilization of energy stored in the form of Na' and K+ ion gradients by bacterial cells, *Eur. J. Biochem.*, 1983, 134:345-349.

Brown C, Gaspar J, Pettit A, Lee R, Gu X, Wang H, Manning C, Voland C, Goldring SR, Goldring MB,Libermann TA, Gravallese EM, Oettgen P., ESE-1 is a novel transcriptional mediator of angiopoietin-1 expression in the setting of inflammation, *J Biol Chem.*, 2004, 279(13):12794-12803.

Burton ER, Gaffar A, Lee SJ, Adeshuko F, Whitney KD, Chung JY, Hewitt SM, Huang GS, Goldberg GL,Libutti SK, Kwon M. Downregulation of filamin A interacting protein 1-like is associated with promoter methylation and induces an invasive phenotype in ovarian cancer. *Mol Cancer Res.*, 2011, 9(8):1126-1138.

Chen Z, Chen L, Baker K, Olszak T, Zeissig S, Huang YH, Kuo TT, Mandelboim O, Beauchemin N, Lanier LL, Blumberg RS., CEACAM1 dampens antitumor immunity by down-regulating NKG2D ligand expression on tumor cells, *J Exp Med.*, 2011, 208(13):2633-2640.

Chevet E, Fessart D, Delom F, Mulot A, Vojtesek B, Hrstka R, Murray E, Gray T, Hupp T., Emerging roles for the pro-oncogenic anterior gradient-2 in cancer development, *Oncogene*, 2013, 32(20):2499-2509.

Chrzanowska-Wodnicka M and Burridge K, Rho-stimulated contractility drives the formation of stress fibers and focal adhesions, *J Cell Biol.*, 1996, 133:1403–1415.

Cornils H, Kohler RS, Hergovich A, Hemmings BA., Human NDR kinases control G(1)/S cell cycle transition by directly regulating p21 stability, *Mol Cell Biol.*, 2011,

31(7):1382-1395.

Debauve G, Capouillez A, Belayew A, Saussez S, The helicase-like transcription factor and its implication in cancer progression, *Cellular and Molecular Life Sciences*, 2008, 65(4):591-604.

DeBerardinis RJ, Lum JJ, Hatzivassilio G, The biology of cancer: metabolic reprogramming fuels cell growth and proliferation, *Cell Metabolism*, 2008, 7(1):11-20.

Duensing S and Münger K, Mechanisms of genomic instability in human cancer: Insights from studies with human papillomavirus oncoproteins, *International Journal of Cancer*, 2004, 109(2):157–162.

Espinosa AM, Alfaro A, Roman-Basaure E, Guardado-Estrada M, Palma Í, Serralde C, Medina I, Juárez E, Bermúdez M, Márquez E, Borges-Ibáñez M, Muñoz-Cortez S, Alcántara-Vázquez A, Alonso P, Curiel-Valdez J, Kofman S, Villegas N, Berumen J., Mitosis is a source of potential markers for screening and survival and therapeutic targets in cervical cancer, *PLoS One*, 2013, 8(2):e55975.

Fico A, De Chevigny A, Egea J, Bösl MR, Cremer H, Maina F, Dono R., Modulating Glypican4 suppresses tumorigenicity of embryonic stem cells while preserving self-renewal and pluripotency, *Stem Cells*, 2012, 30(9):1863-1874.

Fitau J, Boulday G, Coulon F, Quillard T, Charreau B., The adaptor molecule Lnk negatively regulates tumor necrosis factor-alpha-dependent VCAM-1 expression in endothelial cells through inhibition of the ERK1 and -2 pathways, *J Biol Chem.*, 2006, 281(29):20148-20159.

Fujita Y, Hayashi T, Kiyomitsu T, Toyoda Y, Kokubu A, Obuse C, Yanagida M., Priming of centromere for CENP-A recruitment by human hMis18alpha, hMis18beta, and M18BP1, *Dev Cell.*, 2007, 12(1):17-30.

Galeano F, Rossetti C, Tomaselli S, Cifaldi L, Lezzerini M, Pezzullo M, Boldrini R, Massimi L, Di Rocco CM, Locatelli F, Gallo A. ADAR2-editing activity inhibits glioblastoma growth through the modulation of the CDC14B/Skp2/p21/p27 axis. *Oncogene*, 2013,32(8):998-1009.

Grogan TM, Miller P, Nitta H, Na+, K+-ATPase expression in cervical dysplasia and

cancer, US Patent 7,851,145, 2010.

Hammes LS, Tekmal RR, Naud P, Edelweiss MI, Kirma N, et al., Macrophages, inflammation and risk of cervical intraepithelial neoplasia (CIN) progression--clinicopathological correlation. *Gynecol Oncol*., 2007, 105:157-165.

He M, Kratz LE, Michel JJ, Vallejo AN, Ferris L, et al., Mutations in the human SC4MOL gene encoding a methyl sterol oxidase cause psoriasiform dermatitis, microcephaly, and developmental delay. *J Clin Invest.*, 2011, 121: 976-984.

Herdman MT, Pett MR, Roberts I, Interferon-β treatment of cervical keratinocytes naturally infected with human papillomavirus 16 episomes promotes rapid reduction in episome numbers and emergence of latent integrants, *Carcinogenesis*, 2006, 27(11):2341-2353.

Higashi K, Kubo H, Watanabe H, Fujimori K, Mikami T, Kaneko H., Adipokine ganglioside GM2 activator protein stimulates insulin secretion., *FEBS Lett.*, 2011, 585(16):2587-2591.

Hill E, Clarke M, Barr FA, The Rab6-binding kinesin, Rab6-KIFL, is required for cytokinesis, *EMBO J.*, 2000, 19(21):5711-5719.

Hoffman RM, Altered methionine metabolism and transmethylation in cancer, *Anticancer Research*, 1985, 5(1):1-30.

Jin HO, Hong SE, Kim JH, Choi HN, Kim K, An S, Choe TB, Hwang CS, Lee JH, Kim JI, Kim HA, Kim EK,Noh WC, Hong YJ, Hong SI, Lee JK, Park IC., Sustained overexpression of Redd1 leads to Akt activation involved in cell survival, *Cancer Lett.*, 2013, 336(2):319-324.

Johansson K, Järvliden J, Gogvadze V, Morgenstern R., Multiple roles of microsomal glutathione transferase 1 in cellular protection: a mechanistic study, *Free Radic Biol Med.*, 2010, 49(11):1638-1645.

Kanters E, van Rijssel J, Hensbergen PJ, Hondius D, Mul FP, Deelder AM, Sonnenberg A, van Buul JD, Hordijk PL, Filamin B mediates ICAM-1-driven leukocyte transendothelial migration, *J Biol Chem.*, 2008, 283(46):31830-31839.

Kasof GM, Lu JJ, Liu D, Speer B, Mongan KN, Gomes BC, Lorenzi MV, Tumor necrosis factor-alpha induces the expression of DR6, a member of the TNF receptor family,

through activation of NF-kappaB, *Oncogene*, 2001, 20(55):7965-7975.

Keller AD and Maniatis T, Identification and characterization of a novel repressor of beta-interferon gene expression, *Genes Dev.*, 1991, 5:868-879.

Kim DH, Roh YG, Lee HH, Lee SY, Kim SI, Lee BJ, Leem SH, The E2F1 oncogene transcriptionally regulates NELL2 in cancer cells, *DNA Cell Biol.*, 2013, 32(9):517-523.

Koh EH, Cho YW, Mun YJ, Ryu JH, Kim EJ, Choi DS, Maeng KY, Han J, Kang D, Upregulation of human mammaglobin reduces migration and invasion of breast cancer cells, *Cancer Invest.*, 2014, 32(1):22-29.

Kwan HT, Chan DW, Cai PC, Mak CS, Yung MM, Leung TH, Wong OG, Cheung AN, Ngan HY., AMPK activators suppress cervical cancer cell growth through inhibition of DVL3 mediated Wnt/β-catenin signaling activity, *PLoS One*, 2013, 8(1):e53597.

Kwon M, Hanna E, Lorang D, He M, Quick JS, Adem A, et al., Functional characterization of filamin a interacting protein 1-like, a novel candidate for antivascular cancer therapy, *Cancer Res.*, 2008, 68:7332–7341.

Munoz-Pinedo C, Ruiz-Ruiz C, de Almodo´ var CR, Palacios C, Lo´pez-Rivas A, Inhibition of glucose metabolism sensitizes tumor cells to death receptor-triggered apoptosis through enhancement of death-inducing signaling complex formation and apical procaspase-8 processing, *J Biol Chem.*, 2003, 278(15):12759–12768.

Leman AR, Dheekollu J, Deng Z, Lee SW, Das MM, Lieberman PM, Noguchi E, Timeless preserves telomere length by promoting efficient DNA replication through human telomeres, *Cell Cycle*, 2012, 11(12):2337-2347.

Le Roux LG and Moroianu J, Nuclear entry of high-risk human papillomavirus type 16 E6 oncoprotein occurs via several pathways. *J Virol.*, 2003, 77(4):2330-2337.

Liu J, Di G, Wu CT, Hu X, Duan H, CEACAM1 inhibits cell-matrix adhesion and promotes cell migration through regulating the expression of N-cadherin, *Biochem Biophys Res Commun.*, 2013, 430(2):598-603.

Liu P, Gou M, Yi T, Qi X, Xie C, Zhou S, Deng H, Wei Y, Zhao X, The enhanced antitumor

effects of biodegradable cationic heparin-polyethyleneimine nanogels delivering HSulf-1 gene combined with cisplatin on ovarian cancer, *Int J Oncol.*, 2012, 41(4):1504-1512.

Liu J, Wang Z, Jiang K, Zhang L, Zhao L, Hua S, Yan F, Yang Y, Wang D, Fu C, Ding X, Guo Z, Yao X., PRC1 cooperates with CLASP1 to organize central spindle plasticity in mitosis, *J Biol Chem.*, 2009, 284(34):23059-23071.

Longoni N, Sarti M, Albino D,, ETS transcription factor ESE1/ELF3 orchestrates a positive feedback loop that constitutively activates NF-κB and drives prostate cancer progression, *Cancer Res.*, 2013, 73(14):4533-4547.

Lung HL, Cheung AK, Cheng Y, Kwong FM, Lo PH, Law EW, Chua D, Zabarovsky ER, Wang N, Tsao SW, Stanbridge EJ, Lung ML, Functional characterization of THY1 as a tumor suppressor gene with antiinvasive activity in nasopharyngeal carcinoma. *Int J Cancer*, 2010, 127(2):304-312.

Manning AL, Ganem NJ, Bakhoum SF, Wagenbach M, Wordeman L, Compton DA, The kinesin-13 proteins Kif2a, Kif2b, and Kif2c/MCAK have distinct roles during mitosis in human cells, *Mol Biol Cell*, 2007, 18(8):2970-2979.

Marshall MJ, Goldberg DM, Neal FE, Millar DR, Enzymes of glucose metabolism in carcinoma of the cervix and endometrium of the human uterus, *Br. J. Cancer*, 1978, 37: 990-1001.

Mnich SJ, Blanner PM, Hu LG, , Critical role for apoptosis signal-regulating kinase 1 in the development of inflammatory K/BxN serum-induced arthritis, Int Immunopharmacol., 2010, 10(10):1170-1176.

Neesse A, Gangeswaran R, Luettges J, Feakins R, Weeks ME, Lemoine NR, Crnogorac-Jurcevic T, Sperm-associated antigen 1 is expressed early in pancreatic tumorigenesis and promotes motility of cancer cells, *Oncogene*, 2007, 26(11):1533-1545.

Oh ST, Kyo S, Laimins LA, Telomerase activation by human papillomavirus type 16 E6 protein: induction of human telomerase reverse transcriptase expression through Myc and GC-rich Sp1 binding sites. *J Virol.*, 2001, 75:5559-5566.

Omu AE, Al-Azemi MK, Omu FE, Fatinikun T, Abraham S, George S, Mahnazhath N,

Butyrylcholinesterase activity in women with diabetes mellitus in pregnancy: correlation with antioxidant activity, *J Obstet Gynaecol.*, 2010, 30(2):122-126.

Ono M and Hakomori S, Glycosylation defining cancer cell motility and invasiveness, *Glycoconjugate Journal*, 2004, 20:71–78.

Patel M, Dalvi P, Gokulgandhi M, Kesh S, Kohli T, Pal D, Mitra AK. Functional characterization and molecular expression of large neutral amino acid transporter (LAT1) in human prostate cancer cells, *Int J Pharm.*, 2013, 443(1-2):245-253.

Peck JW, Oberst M, Bouker KB, Bowden E, Burbelo PD., The RhoA-binding protein, rhophilin-2, regulates actin cytoskeleton organization, *J Biol Chem.*, 2002, 277(46):43924-43932.

Pellerin L, Magistretti PJ, Excitatory amino acids stimulate aerobic glycolysis in astrocytes via an activation of the Na+/K+ ATPase, *Dev Neurosci.*, 1996, 18:336-342.

Peters HL, Tuli A, Sharma M, Naslavsky N, Caplan S, MacDonald RG, Solheim JC, Regulation of major histocompatibility complex class I molecule expression on cancer cells by amyloid precursor-like protein 2, *Immunol Res.*, 2011, 51(1):39-44.

Petrovic I, Kovacevic-Grujicic N, Stevanovic M, ZBP-89 and Sp3 down-regulate while NF-Y up-regulates SOX18 promoter activity in HeLa cells, *Mol Biol Rep.*, 2009, 36(5):993-1000.

Randle PJ, Regulatory Interactions between Lipids and Carbohydrates: The Glucose Fatty Acid Cycle After 35 Years, *Diabetes/Metabolism Reviews*, 1998, 14(4): 263–283.

Razmara M, Srinivasula SM, Wang L, Poyet JL, Geddes BJ, DiStefano PS, Bertin J, Alnemri ES. CARD-8 protein, a new CARD family member that regulates caspase-1 activation and apoptosis. *J Biol Chem.*, 2002, 277(16):13952-13958.

Ritzman AM, Hughes-Hanks JM, Blaho VA, The chemokine receptor CXCR2 ligand KC (CXCL1) mediates neutrophil recruitment and is critical for development of experimental Lyme arthritis and carditis, *Infect. Immun.*, 2010,

78(11):4593-4600.

Ruutu M, Peitsaro P, Johansson B, Syrjänen S, Transcriptional profiling of a human papillomavirus 33–positive squamous epithelial cell line which acquired a selective growth advantage after viral integration, *Int. J. Cancer*, 2002, 100: 318-326.

Ryu J, Park SG, Lee PY, Cho S, Lee do H, Kim GH, Kim JH, Park BC, Dimerization of pro-oncogenic protein Anterior Gradient 2 is required for the interaction with BiP/GRP78, *Biochem Biophys Res Commun.*, 2013, 430(2):610-615.

Sailaja GS, Bhoopathi P, Gorantla B, Chetty C, Gogineni VR, Velpula KK, Gondi CS, Rao JS, The secreted protein acidic and rich in cysteine (SPARC) induces endoplasmic reticulum stress leading to autophagy-mediated apoptosis in neuroblastoma. *Int J Oncol.*, 2013, 42(1):188-196.

Sarkar AK, Tortolero-Luna G, Follen M, Sastry KJ, Inverse correlation of cellular immune responses specific to synthetic peptides from the E6 and E7 oncoproteins of HPV-16 with recurrence of cervical intraepithelial neoplasia in a cross-sectional study, *Gynecologic Oncology*, 2005, 99:S251-S261.

Schmid S, Bieber M, Zhang F, Zhang M, He B, Jablons D, Teng NN., Wnt and hedgehog gene pathway expression in serous ovarian cancer, *Int J Gynecol Cancer.*, 2011, 21(6):975-980.

Sekine H, Takahashi M, Iwaki D, Fujita T, The role of MASP-1/3 in complement activation, *Adv Exp Med Biol.*, 2013, 735:41-53.

Serva A, Knapp B, Tsai YT, Claas C, Lisauskas T, Matula P, Harder N, Kaderali L, Rohr K, Erfle H, Eils R,Braga V, Starkuviene V, miR-17-5p regulates endocytic trafficking through targeting TBC1D2/Armus, *PLoS One*, 2012, 7(12):e52555.

Shiizaki S, Naguro I, Ichijo H., Activation mechanisms of ASK1 in response to various stresses and its significance in intracellular signaling, *Adv Biol Regul.*, 2013, 53(1):135-144.

Silverman RH, Viral Encounters with 2′,5′-Oligoadenylate Synthetase and RNase L during the Interferon Antiviral Response, *J Virol.*, 2007, 81(23):12720-12729.

Simonart T, Boelaert JR, Mosselmans R, Andrei G, Noel JC, De Clercq E, Snoeck R,

Antiproliferative and apoptotic effects of iron chelators on human cervical carcinoma cells, *Gynecologic Oncology*, 2002, 85:95–102.

Stolle K, Schnoor M, Fuellen G, Spitzer M, Cullen P, Lorkowski S, Cloning, genomic organization, and tissue-specific expression of the RASL11B gene, *Biochim Biophys Acta.*, 2007, 1769(7-8):514-524.

Suen PM, Zou C, Zhang YA, Lau TK, Chan J, Yao KM, Leung PS., PDZ-domain containing-2 (PDZD2) is a novel factor that affects the growth and differentiation of human fetal pancreatic progenitor cells, *Int J Biochem Cell Biol.*, 2008,40(4):789-803.

Tanaka Y, Fujii M, Hayashi K, Chiba N, Akaishi T, Shineha R, Nishihira T, Satomi S, Ito Y, Watanabe T, Satake M, The chimeric protein, PEBP2 beta/CBF beta-SMMHC, disorganizes cytoplasmic stress fibers and inhibits transcriptional activation, Oncogene, 1998, 17(6):699-708.

Textor S, D€urst M, Jansen L, Activating NK cell receptor ligands are differentially expressed during progression to cervical cancer, *Int J Cancer*, 2008, 123:2343-2353.

Thakur HC, Singh M, Nagel-Steger L, Prumbaum D, Fansa EK, Gremer L, Ezzahoini H, Abts A, Schmitt L,Raunser S, Ahmadian MR, Piekorz RP, Role of centrosomal adaptor proteins of the TACC family in the regulation of microtubule dynamics during mitotic cell division, *Biol Chem.*, 2013, 394(11):1411-1423.

To S, Rodda SJ, Rathjen PD, Keough RA, Modulation of CP2 family transcriptional activity by CRTR-1 and sumoylation, *PLoS One*, 2010, 5(7):e11702.

Tzeng HE, Tsai CH, Chang ZL, Su CM, Wang SW, Hwang WL, Tang CH, Interleukin-6 induces vascular endothelial growth factor expression and promotes angiogenesis through apoptosis signal-regulating kinase 1 in human osteosarcoma, *Biochem Pharmacol*., 2013, 85(4):531-540.

Uno K, Kuwabara H, Terado Y, Kojima K, Kawakami T, Kamma H, Sakurai H, Sakamoto A, Kurata A, Divergent expression of L-type amino acid transporter 1 during uterine cervical carcinogenesis, *Hum Pathol.*, 2011, 42(11):1660-1666.

Vinokurova S, et al. Clonal history of papillomavirusinduced dysplasia in the female

lower genital tract, *J Natl Cancer Inst.*, 2005, 97:1816-1821.

Wandall HH, Irazoqui F, Tarp MA, Bennett EP, Mandel U, Takeuchi H, Kato K, Irimura T, Suryanarayanan G, Hollingsworth MA, Clausen H, The lectin domains of polypeptide GalNAc-transferases exhibit carbohydrate-binding specificity for GalNAc: lectin binding to GalNAc-glycopeptide substrates is required for high density GalNAc-O-glycosylation, *Glycobiology*, 2007, 17(4):374-387.

Wang J, Zhao J, Chu ES, Mok MT, Go MY, Man K, Heuchel R, Lan HY, Chang Z, Sung JJ, Yu J, Inhibitory role of Smad7 in hepatocarcinogenesis in mice and in vitro, *J Pathol.*, 2013, 230(4):441-452.

Woodman CBJ, Stuart I. Collins SI, Young LS, The natural history of cervical HPV infection: unresolved issues, *Nature Reviews*, 2007, 7:11-22.

Xing C, Xie H, Zhou L, Zhou W, Zhang W, Ding S, Wei B, Yu X, Su R, Zheng S, Cyclin-dependent kinase inhibitor 3 is overexpressed in hepatocellular carcinoma and promotes tumor cell proliferation, *Biochem Biophys Res Commun.*, 2012, 420(1):29-35.

Yang GF, Chao K, Li XM, Rao HL, Deng HX, Wu HM, Xie D, Significance of expression of THY1 protein in epithelial ovarian cancer, *Zhonghua Zhong Liu Za Zhi*, 2009, 31(3):203-207.

Yu Y, Munger K., Human papillomavirus type 16 E7 oncoprotein inhibits the anaphase promoting complex/cyclosome activity by dysregulating EMI1 expression in mitosis, *Virology*, 2013, 446(1-2):251-259.

Zhang JL, Chen GW, Liu YC, Wang PY, Wang X, Wan YL, Zhu J, Gao HQ, Yin J, Wang W, Tian ML, Secreted protein acidic and rich in cysteine (SPARC) suppresses angiogenesis by down-regulating the expression of VEGF and MMP-7 in gastric cancer, *PLoS One*, 2012, 7(9):e44618.

Zheng H, Li Q, Chen R, Zhang J, Ran Y, He X, Li S, Shu HB, The dual-specificity phosphatase DUSP14 negatively regulates tumor necrosis factor- and interleukin-1-induced nuclear factor-κB activation by dephosphorylating the protein kinase TAK1, *J Biol Chem.*, 2013, 288(2):819-825.

Zheng Y, Yang W, Aldape K, He J, Lu Z, Epidermal growth factor (EGF)-enhanced

vascular cell adhesion molecule-1 (VCAM-1) expression promotes macrophage and glioblastoma cell interaction and tumor cell invasion, *J Biol Chem.*, 2013, 288(44):31488-31495.

Zhou Y, Hagood JS, Lu B, Merryman WD, Murphy-Ullrich JE, Thy-1-integrin alphav beta5 interactions inhibit lung fibroblast contraction-induced latent transforming growth factor-beta1 activation and myofibroblast differentiation, *J Biol Chem.*, 2010, 285(29):22382-22393.
